# The effect of anthocyanin from *Dioscorea alata* L. after purification, identification on antioxidant capacity in mice

**DOI:** 10.1002/fsn3.3547

**Published:** 2023-07-18

**Authors:** Pingfei Qiu, Junpu Chen, Junlong Wu, Qin Wang, Yanrong Hu, Xiaochun Li, Huiyu Shi, Xuemei Wang

**Affiliations:** ^1^ Animal Nutrition, Reproduction and Breeding Laboratory, School of Animal Science and Technology Hainan University Haikou China

**Keywords:** anthocyanin, antioxidant capacity, *Dioscorea alata* L., macroporous

## Abstract

Increasing findings devote to searching for natural active compositions as additives to ameliorate health status. Anthocyanin, water‐soluble natural pigment, has been concerned due to its favorable antioxidant activity. In this study, we purified anthocyanin from *Dioscorea alata* L., identified its compounds, and evaluated its antioxidant properties. The results indicated that the purity of anthocyanin increased to 39.59 ± 1.56%, 60.18 ± 1.97%, and 81.08 ± 1.97% after purification via AB‐8 macroporous resin, Sep‐Pak C18 solid phase, and LH‐20 Sephadex stepwise. Ultra‐performance liquid chromatography tandem mass spectrometer results indicated that paeoniflorin‐3,5‐O‐dihexoside, petunin‐3‐O‐feruloyl‐glucoside‐5‐O‐glucoside, cyanidin‐3‐O‐feruloyl glucoside‐5‐O‐glucoside, cyanidin‐3‐O‐sophoroside, and petunin‐3,5‐O‐dihexoside were the major compounds. The purified anthocyanin exhibited stronger antioxidant activity than the unpurified extract and ascorbic acid, whereas weaker than that of cyanidin‐3‐O‐glucoside in general, which was assessed using DPPH, ABTS, and Fe^3+^ reducing capacity methods. Moreover, the purified anthocyanin increased GSH‐Px, total antioxidant capacity, and superoxide dismutase activities and decreased malondialdehyde concentration on serum in mice after administering lipopolysaccharide for 24 h (*p* < .05). To summarize, the purified anthocyanin boasts more outstanding antioxidant properties than that of crude extracts. These results provide a reference with source of anthocyanin and it is conducive to use *Dioscorea alata* L. resources.

## Graphical abstract

In our study, the purity of anthocyanin increased to 39.59 ± 1.56%, 60.18 ± 1.97%, and 81.08 ± 1.97% after purification via AB‐8 macroporous resin, Sep‐Pak C18 solid phase, and LH‐20 Sephadex stepwise. Ultra‐performance liquid chromatography tandem mass spectrometer results indicated that paeoniflorin‐3,5‐O‐dihexoside, petunin‐3‐O‐feruloyl‐glucoside‐5‐O‐glucoside, cyanidin‐3‐O‐feruloyl glucoside‐5‐O‐glucoside, cyanidin‐3‐O‐sophoroside, and petunin‐3,5‐O‐dihexoside were the major compounds. The purified anthocyanin exhibited stronger antioxidant activity than the unpurified extract and ascorbic acid, whereas weaker than that of cyanidin‐3‐O‐glucoside in general, which was assessed using DPPH, ABTS, and Fe3+ reducing capacity methods and the purified anthocyanin increased GSH‐Px, total antioxidant capacity, and superoxide dismutase activities and decreased malondialdehyde concentration on serum in mice after administering lipopolysaccharide for 24 h (*p* < .05).

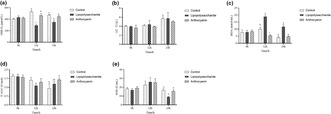

## 1 INTRODUCTION

*Dioscorea alata* L. (Dioscoreaceae, DL), also known as winged yam, is widely planted in the tropics or subtropics (Korada & Edison, 2010). DL is the second largest food crops just less than cassava in some countries due to rich nutrition, delicious taste, and bright color (Arnau et al., 2009). Several previous reports suggested that many kinds of vitamins were found in DL, including thiamin, riboflavin (Li et al., 2009), and it can be used for relieving diarrhea and tonifying spleen (Lou, 2013). In addition, some active components, including anthocyanidin and dietary fiber, can be extracted from DL (Zhong, 2010).

Anthocyanin, polyphenolic water‐soluble pigments, that is polyhydroxy and polymethoxy derivatives of 2‐phenylbenzopyrylium salts has been given more attention for its antioxidant capacity that is conducive to limit the overproduction of reactive oxygen species (Bräunlich et al., 2013; Lee et al., 2017; Lim et al., 2013). Up to now more than 600 anthocyanins have been found (He & Giusti, 2010) and pelargonidin, cyanidin, delphinidin, peonidin, petunidin, malvidin, and their derivatives are common anthocyanins in plants. Delphinidin boasted the most active scavenger against superoxide anion among these (Tsuda et al., 1996). In addition, many studies reported that anthocyanin protects cell lines against oxidative stress (Heo & Lee, 2005; Isaak et al., 2017). Not surprisingly, the antioxidant functions of anthocyanin have been reported. For example, a previous review involved in cardiovascular disease indicated that anthocyanin may modulate several signaling pathways (Wallace, 2011), which are more or less related to potent antioxidant capacity of anthocyanin (He & Giusti, 2010). In addition, cyanidin‐3‐glucoside ameliorated hepatic ischemia–reperfusion induced by oxidative stress in rats (Tsuda et al., 2000) and some anthocyanins, such as cyanidin, delphinidin, and malvidin, induced upregulation of antioxidant response element pathways to some extent (Thoppil et al., 2012).

A host of researchers devoted to extracting anthocyanin from various plants, due to its potent antioxidant property and biological potential to development of healthcare products (Silva et al., 2017). Fruit is a critical tissue that deposits anthocyanin (Silva et al., 2017) and mulberry fruits especially the mulberry cultivar J33 showed the highest amount (Xie et al., 2019). A previous study found that anthocyanin can be extracted from black rice and the maximum yield can take upwards of 266 mg/100 g (Yi et al., 2021). Surprisingly, several researchers successfully extracted anthocyanin from onion peel and such extracted anthocyanin can be used as natural functional food additives (S et al., 2022). Many studies also reported that anthocyanins can be extracted from other plants, such as red cabbage, sweet cherry, cricket vine through solid–liquid extraction and supercritical fluid extraction (Chandrasekhar et al., 2012; Oancea et al., 2013; Paula et al., 2014). However, there are many impurities in anthocyanins preliminarily processed, including polysaccharide, amino acid, organic acid (Jampani et al., 2014).

Macroporous resin, economical and common method used for purifying anthocyanin, can separate impurities from target substances, which lays a foundation for further qualitative analysis. High‐performance liquid chromatography, ultra‐performance liquid chromatography tandem mass spectrometer (UPLC‐MS), and UV–visible spectrometry can be used to investigate anthocyanin. To investigate more anthocyanin resources and enhance the utility value of purple ginseng potato, herein, we purified anthocyanin from DL via macroporous resin, solid phase extraction, and sephadex stepwise, and UPLC‐MS technique was successfully used for analyzing bioactive compounds. Finally, DPPH, ABTS, FRAP methods, and mice experiment were used for evaluating the antioxidant capacity in vitro and in vivo. The result of this research will provide a reference for source of anthocyanins and lay the foundation for subsequent researches.

## 2 MATERIALS AND METHODS

DL used in this study was purchased from Hainan University Farm (Hainan, China) and stored at room temperature away from light prior to use. Briefly, after the surface soil was rinsed, winged yam was sliced and dried at 45°C. Afterwards, dried winged yam was ground using a grinder and sieved through a 40‐mesh sieve. The powder is stored at −20°C before being used for extraction.

### 2.1 Materials and reagents

Ascorbic acid was purchased from Regent Chemicals Co. LTD (Tianjin, China). Standard product of anthocyanin (cyanidin‐3‐O‐glucoside) was purchased from Yuanye Biotechnology Co., LTD (Shanghai, China). All the kits associated with antioxidant property evaluation were purchased from Nanjing Jiancheng Institute of Biological Engineering (Jiangsu, China). AB‐8 macroporous resin, C18 solid phase extraction column, and LH‐20 sephadex were purchased from Dongpeng Chemical Co., LTD (Jiangxi, China), Waters (USA), and Yuanye Biotechnology Co., LTD (Shanghai, China) respectively. All chemical reagents were purchased from Xilong Chemical Co. LTD (Guangdong, China).

### 2.2 Anthocyanin purification

#### 2.2.1 AB‐8 macroporous resin purification

Anthocyanin was extracted in our previous study in the condition of 19 min of ultrasound assistance, a liquid‐to‐solid ratio of 1:22 (g/mL), and hydrochloric acid volume fraction of 0.63% (Chen et al., 2022). In this experiment, AB‐8 macroporous resin was used to purify anthocyanin after the extracting solution was concentrated by a rotary evaporator (BUCHI V‐850, Switzerland). Before use, macroporous resin was pretreated according to the previously described methods (Lin et al., 2012; Wang et al., 2019).

#### 2.2.2 Static adsorption and desorption kinetic curve

After activation, 1 g of resin was added into 20 mL concentrated anthocyanin extract in a conical flask and the mixture was placed in a shaker (BWS‐10, Shanghai YiHENG SCIENTIFIC Instrument Co., LTD, Shanghai, China) under 30°C at 80 rpm for 6 h. During adsorption, the supernatant collected per hour was applied to detect absorbance, and resin carrying anthocyanin was collected for desorption. Twenty milliliters of 60% ethanol was added into the flask to immerse resin which were incubated under 30°C at 80 rpm for 6 h. Similarly, the supernatant was collected each hour to determine absorbance. Adsorption and desorption rate were calculated according to Equations (1) and (2). The optimal adsorption and desorption time were selected to assay the suitable purified time:

(1) $$\text{Adsorption rate}=\frac{A_{0}-A_{1}}{A_{0}}\times 100\%$$

(2) $$\text{Desorption rate}=\frac{A_{3}}{A_{2}-A_{0}}\times 100\%$$

where *A*_0_ is the absorbance of anthocyanin extract before adsorption at 528 nm, *A*_1_ is the absorbance of anthocyanin's extract at 528 nm at different time points, *A*_2_ is the absorbance anthocyanin extract before desorption, and *A*_3_ is the absorbance of anthocyanin extract at 528 nm at different time points (1, 2, 3, 4, 5, and 6 h).

#### 2.2.3 Effect of pH and extract flow velocity on adsorption efficiency

The effect of pH on adsorption efficiency was investigated at 2.0, 3.0, and 4.0, respectively. The extract flow velocity was set to 2.0 mL/min during the process. The effect of extract flow velocity on adsorption efficiency was investigated at 2.0, 4.0, and 6.0 mL/min, respectively. The pH was set to 3.0 during the process.

Resin AB‐8 (1 g) was added into 20 mL concentrated anthocyanin extract and incubated under 30°C at 80 rpm for 1–5 h in aforementioned single‐factor experiments. The adsorption efficiency was calculated according to Equation (1).

#### 2.2.4 Effect of concentration and flow velocity on anthocyanin content in eluent

Ethanol/water mixtures at different ratios (40%, 60%, and 80%) were used as solvents to determine the influence of eluent on desorption efficiency. The eluent flow velocity was set to 2.0 mL/min. The effect of eluent flow velocity on desorption efficiency was explored with 2.0, 4.0, and 6.0 mL/min, respectively. The eluent concentration was set to 60%. One tube of the desorption solution was collected per half hour for analyses. Anthocyanin content was calculated according to Equation (3), which found that there is a linear relation between absorbance and anthocyanin content (Chen et al., 2022):

(3) $$\text{Anthocyanin content}\;\left(\mathrm{mg}/\mathrm{mL}\right)=26.54\times A_{4}–0.03075$$

where A_4_ is the absorbance of collected eluent.

#### 2.2.5 Sep‐Pak C18 and LH‐20 sephadex purification

C18 solid phase extraction column was used to further purify further after purification with AB‐8 resin. The anthocyanin purified by C18 was eluted using methanol and followed lyophilization. Ulteriorly, 0.5 mg/mL of anthocyanin solution (20 mL), a mixture of ultrapure water and freeze‐dried powder purified by C18, was purified using LH‐20 sephadex and methanol/water mixture at different ratios (20%, 35%, and 50%) were applied for stepwise elution (flow velocity 1.0 mL/min). The eluant was collected, freeze‐dried, and stored at −20°C to further analysis after purification each time.

#### 2.2.6 Purity calculation of anthocyanin

Freeze‐dried anthocyanin powder of 5 mg was dissolved in 5 mL ultrapure water and the absorbances were determined after acidic buffers (chloride buffer pH of 1.0, acetate buffer pH of 4.5) were utilized to dilute the anthocyanin solution, respectively. Purity calculation of anthocyanin was calculated according to Equation (4):

(4) $$\text{Purity}\;\left(\%\right)=\frac{A\times M\times \mathrm{DF}\times V}{\varepsilon \times l\times m}\times 100\%$$

where A = (A pH 1.0_520 nm_–A pH 1.0_700 nm_)–(A pH 4.5_520 nm_–A pH 4.57_00 nm_); M is molecular weight of cyanidin‐3‐O‐glucoside chloride, 449.2 g/mol; DF is the dilution factor; V is the volume of anthocyanin solution; ε is the molar absorbance of cyanidin‐3‐O‐glucoside chloride, 26,900 L/(mol/cm); l is the optical distance, 1 cm; m is the mass of anthocyanin, 5 mg.

### 2.3 UPLC‐MS analysis

One milliliter of 5% formic acid is used as solvent to dissolve 100 mg anthocyanin powder in Eppendorf tubes, and the solution was placed on ice for 30 min and centrifuged under 4°C at 12,000 rpm for 10 min to collect supernate for freeze‐drying. The freeze‐dried powder was dissolved in 0.2 mL of 5% formic acid again and the solution was centrifuged under 4°C, at 12,000 rpm for 10 min to collect supernate for further analysis.

The composition of anthocyanin was analyzed by UPLC‐MS. An Vanquish mass spectrometer (Thermo Fisher Scientific Co., LTD, Shanghai, China) with ACQUITY ultra‐high‐performance liquid chromatogramy was used. Chromatogramy was conducted on Waters HSS T3 column (1.8 μm; 100 × 2.1 mm). The UPLC was as follows: Solvent A was composed of 0.1% formic acid and 99.9% acetonitrile and solvent B was composed of water with 0.1% formic acid. The elution was complete using the following gradient: 0–12.0 min 10% A, 90.0% B; 12.0–18.0 min 60% A, 40% B; and 18.0–26.0 min 10% A, 90% B. The injection volume was 2 μL, flow rate was 0.3 mL/min, and column temperature was 40°C. For the identification of anthocyanins, electrospray ionization (ESI) was operated in the positive mode using Q Exactive high‐resolution mass spectrometry detection system (Thermo Fisher Scientific Co., LTD, Shanghai, China) with ESI and Xcalibur workstation. The optimized mass spectrometry conditions were as follows: sheath gas 40, auxiliary gas 10, ion spray voltage +3000 V, temperature 350°C, and ion transfer tube temperature 320°C.

### 2.4 Antioxidant activity detection

DPPH, FRAP, and ABTS methods were applied to detect antioxidant activity of anthocyanin (after LH‐20 purification). In addition, anthocyanin's crude extract, ascorbic acid, and cyanidin‐3‐O‐glucoside were used as controls. Powder of 2 mg was accurately weighed to adjust the concentration as 1 mg/mL, 0.5 mg/mL, 0.25 mg/mL, 0.125 mg/mL, and 0.0625 mg/mL for further determination.

To investigate whether the anthocyanin possess prominent antioxidant effect in vivo, animal experiment was conducted. In all, 36 C57BL/6 male mice with initial body weight of 13.5 ± 1.5 g were purchased from SLAC Laboratory Animal Co. Ltd. (Shanghai, China) fed in the Laboratory Animal Center, Hainan University at 20°C–24°C, relative humidity 40%–50%, and lighting 12 h every day (8:00–20:00). The animal experiment was reviewed and approved by the Ethical Committee of the Hainan University (Haikou, China, Permit number: HNUAUCC‐2022‐00032). After 1 week of acclimation, mice were fasted for 12 h, weighed and randomly divided into three treatments with three cages of four mice per treatment (control group, lipopolysaccharide group: sterile saline + lipopolysaccharide, anthocyanin group: anthocyanin + sterile saline). Anthocyanin (purity 81.08%) and lipopolysaccharide were dissolved in sterile saline at 0.4 mg/mL and 0.2 mg/mL, respectively. Anthocyanin solution or sterile saline was administered orally for 3 days with 10 mg/kg body weight. On Day 4, lipopolysaccharide solution or sterile saline was introduced to mice by intraperitoneal injection after anesthetization with 5 mg/kg body weight. Blood samples were collected via eye sockets at 0 h, 12 h, and 24 h after lipopolysaccharide administration. The samples were placed on ice for 1 h and centrifuged under 4°C at 3000 rpm for 10 min to collect supernatant for further analysis.

### 2.5 Statistical analysis

To compare treatments, statistical analysis was conducted using SPSS 21.0 and significant differences were verified by one‐way ANOVA with Duncan's multiple range test (*p* < .05). All graphs were drawn using Graphpad Prism 8.0. All assays were repeated three times.

## 3 RESULTS AND DISCUSSION

### 3.1 Static adsorption and desorption kinetic curve

**FIGURE 1:**
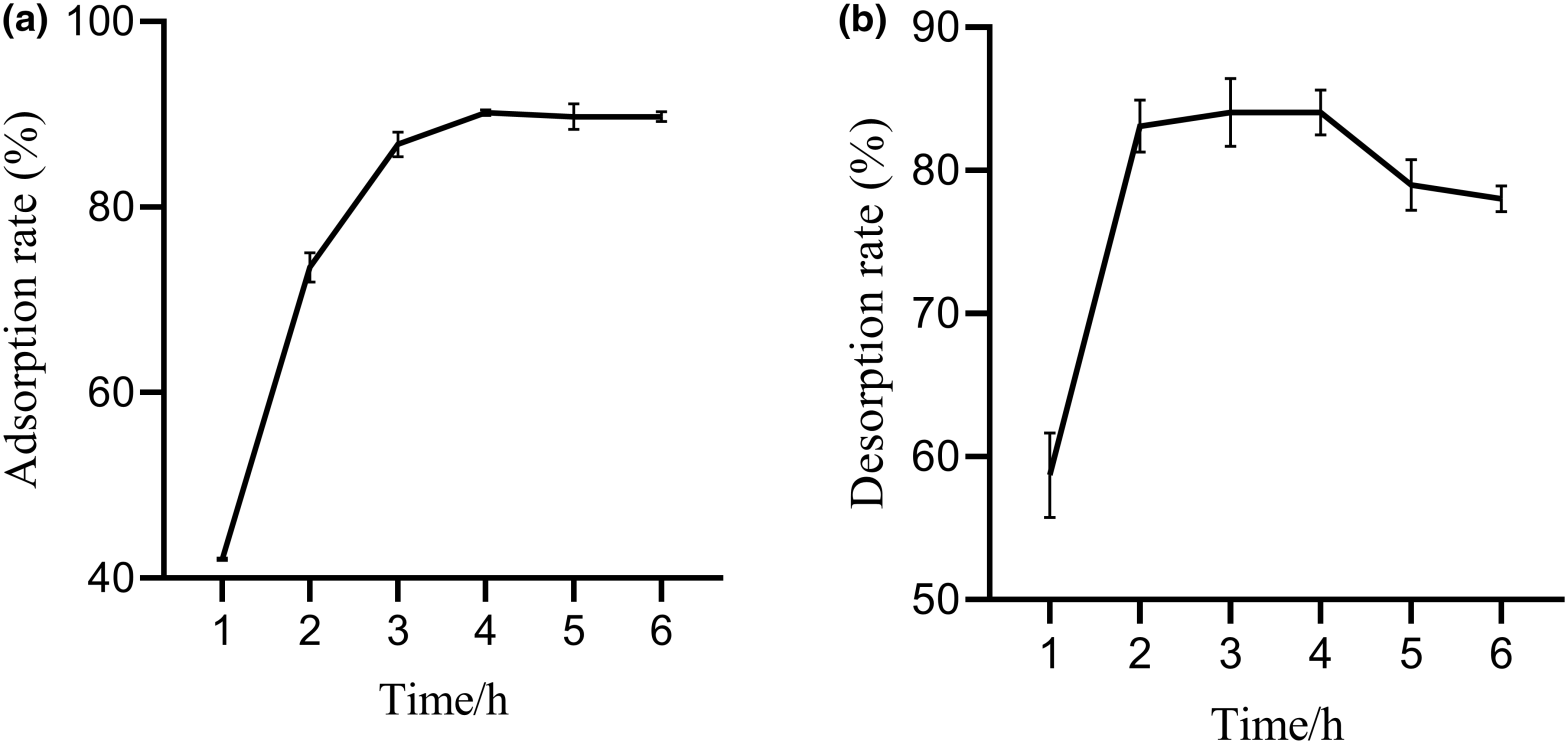
(a) Static adsorption kinetic curve of anthocyanin from *Dioscorea alata* L. (DL) with 1 g AB‐8 resin under 30°C at 80 rpm for 6 h. (b) Static desorption kinetic curve of AB‐8 resin (1 g) adsorbing anthocyanin from DL with 60% ethanol as eluent under the condition of 30°C at 80 rpm for 6 h. Vertical bars represent the standard deviation of each value. All data are presented as mean ± standard deviation (SD).

As shown in Figure 1a, adsorption rate increased with time until a plateau was reached. A sharp increase was observed at the first 3 h and it tended to balance at the last 3 h, where the adsorption rate was as high as 86.75%. The desorption rate could be described as a fast stage followed by a slow desorption, which achieved a plateau at 3rd hour (desorption rate reached 84.06%) (Figure 1b). As a result, the optimal adsorption and desorption time were 4 and 3 h, respectively.

### 3.2 Effect of pH and flow velocity on adsorption rate

**FIGURE 2:**
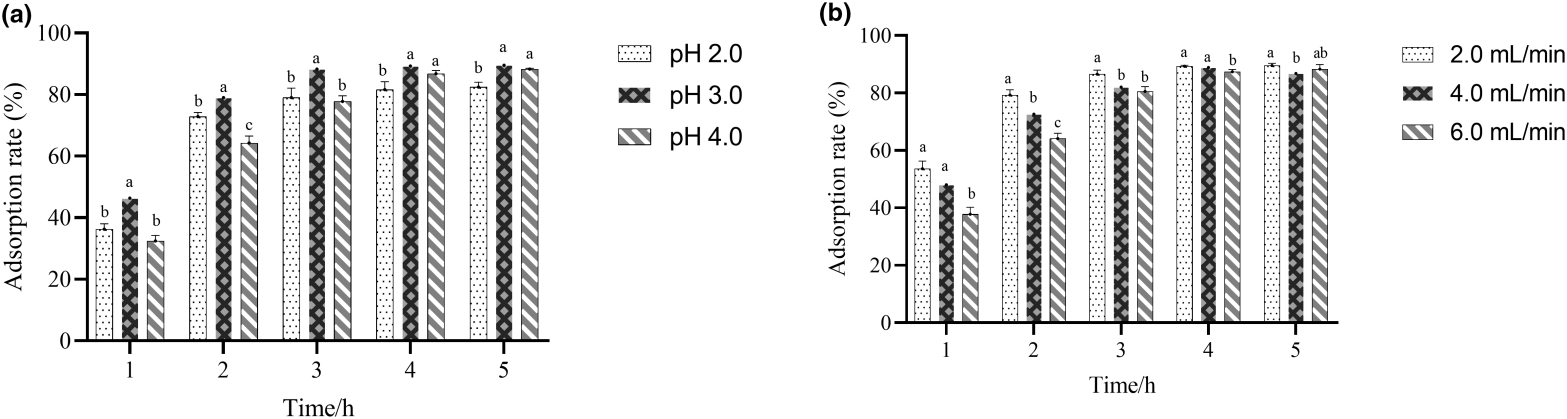
(a) Effect of AB‐8 resin with different pH (2.0, 3.0, 4.0) on anthocyanin adsorption rate. (b) Effect of flow velocity (2.0, 4.0, 6.0 mL/min) on anthocyanin adsorption rate. All data are presented as mean ± SD. ^a, b, c^Within a single time point, there is no statistical difference among the same letter (*p* < .05).

Figure 2a shows that adsorption rate increased with time and a significant difference could be observed between pH 3.0 and pH 2.0, 4.0 at the first 3 h (*p* < .05). The results suggested that pH 3.0 always has a higher adsorption rate at the same point in time and the maximum adsorption rate occurred at 3 h with pH 3.0. As shown in Figure 2b, there is an increase with time at different flow velocity followed by a stable stage. Compared to higher flow velocity (4.0, 6.0 mL/min), the adsorption rate of the lower flow velocity is significantly higher at the first 3 h (*p* < .05). It has been reported that anthocyanin molecules possess strong stability under low pH environment (Março & Scarminio, 2007) but we found that it decreased adsorption rate when pH was lower than 2.0 or higher than 4.0. The results indicated the faster flow velocity the lower adsorption rate, which may be responsible for shorter contact time between anthocyanin and resin. Thus, the optimal pH and flow velocity were 3.0 and 2 mL/min, respectively.

### 3.3 Effect of volume fraction of ethanol and flow velocity on anthocyanin content in eluant

**FIGURE 3:**
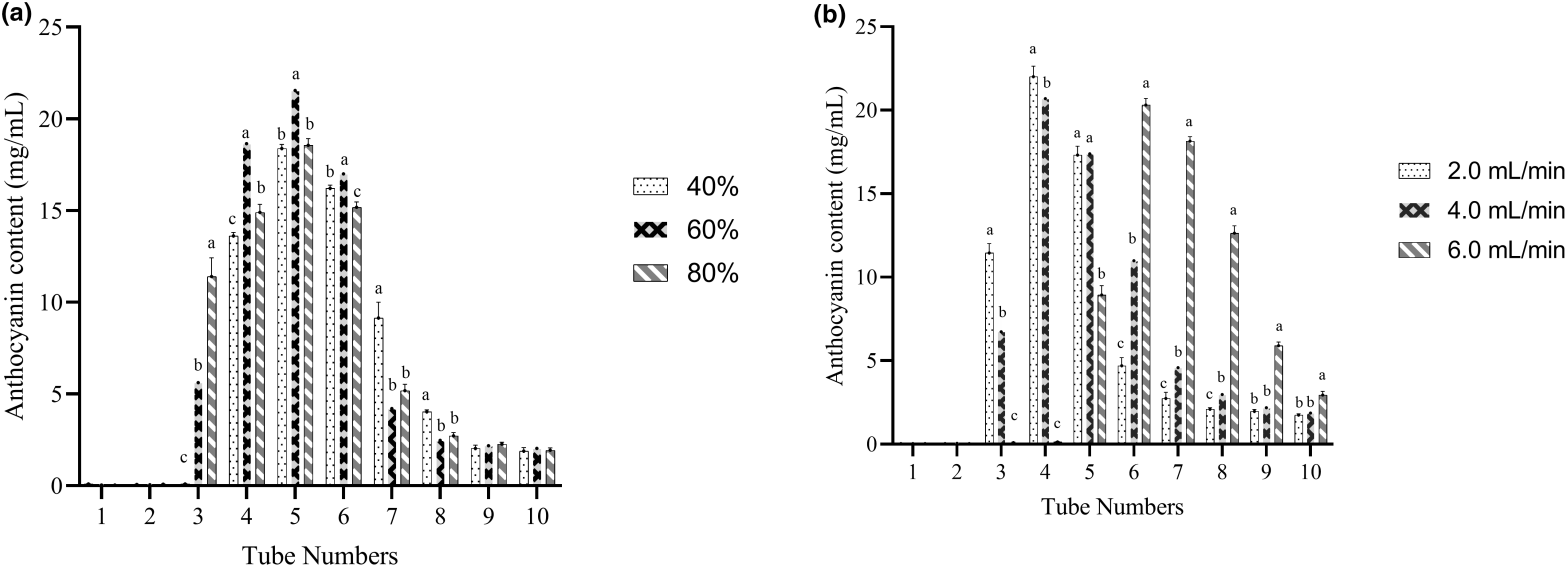
(a) Effect of volume fraction of ethanol on anthocyanin content in the eluent. (b) Effect of flow velocity on anthocyanin content in the eluent. All data are presented as mean ± SD. ^a, b, c^Within a single time point, there is no statistical difference among the same letter (*p* < .05).

With the increase in collected tube numbers, anthocyanin content increased and reached a maximum value of 21.54 mg/mL with a 60% ethanol solution at 5th tube (Figure 3a). Afterwards, a downward trend appeared with the increase in tube numbers. In a previous study, it deemed the best matching of polarity between solvent and adsorbate in 60% ethanol (Wang et al., 2019), which was confirmed by our study again. Generally speaking, although the low flow rate is conducive to anthocyanin collection, it takes a long time. Herein, to obtain anthocyanin efficiently, the flow rate of elution was also investigated. Figure 3b indicated that anthocyanin content reached a maximum value of 22.01 mg/mL with the flow velocity for 2 mL/min at the fourth tube. Moreover, the flow velocity for 6 mL/min showed a lagged trend and anthocyanin content is higher in 6 mL/min at sixth tube, compared to other flow velocity.

### 3.4 Comparison of purity for anthocyanin after each purification

As shown in Table 1, compared to crude anthocyanin extract, anthocyanin purity has increased to 39.59%, 60.18%, and 81.08% after purification for three times, which suggested that impurity could be removed effectively via AB‐8, C18, and LH‐20 purification.

**TABLE 1:** Comparison of Purity for Anthocyanin after Purification.

Purification stages	Purity (%)
Crude extract	5.33 ± 0.15
AB‐8 purification	39.59 ± 1.56
Sep‐Pak C18 purification	60.18 ± 1.97
Sephadex LH‐20 purification	81.08 ± 1.97

### 3.5 UPLC‐MS analysis

As seen in Table 2, it is found 13 anthocyanin components, in which five compounds' ratio is greater than 2% and these were chosen to carry out further identification, namely peaks 4, 7, 8, 9, 11. Herein, the area ratio of peak 7, the largest proportion, reached 76.77% (the chromatograms of anthocyanin UPLC‐MS are shown in Data S1: three replicates of the sample were carried out and the concentrations of standard substance are 1, 10, 50, 100, 200, 300, 500, 800, 1000, 2000 ng/mL).

**TABLE 2:** Identification of anthocyanin compounds from *Dioscorea alata* L. by UPLC‐MS.

Peak	Real time/min	Peak area	Peak area ratio/%	Peak height
1	7.82	2,966,850.377	0.01%	832,769
2	7.93	249,148,015.9	0.76%	25,696,574
3	7.98	253,871,010.1	0.77%	67,242,928
4	8.45	710,461,263.6	2.16%	60,601,779
5	8.59	106,876,159.4	0.32%	23,157,661
6	8.97	152,549,919.1	0.46%	20,173,360
7	9.04	25,299,867,876	76.77%	3,319,315,552
8	9.37	3,829,956,380	11.62%	378,527,868
9	10.15	1,334,711,044	4.05%	349,958,241
10	11.17	158,420,325.1	0.48%	41,531,073
11	11.53	762,843,536.5	2.31%	210,515,633
12	11.58	16,469,815.47	0.05%	5,051,579
13	12.55	78,233,071.7	0.24%	23,242,682

Table 3 summarized the list of compounds and their main fragments observed during UPLC‐MS analyses. Peak 4 showed m/z 625.18, with characteristic fragments at m/z 463.12 and 301.17, which were formed by the loss of hexosyl group. Therefore, it was identified as paeoniflorin‐3, 5‐O‐dihexoside that lost two hexose molecules forming paeoniflorin ion with m/z 301.17. The molecular ion of peak 7 was observed at m/z 817.22 with characteristic fragments at m/z 655.17. Herein, fragment ion of m/z 162 resulted from the elimination of hexose from the molecular ion. This component was identified as petunin‐3‐O‐feruloyl‐glucoside 5‐O‐glucoside based on database comparison and analyses. The parent ion of peak 8 was found at m/z 787.21, with loss fragment ions at m/z 162, 176, indicating loss of hexosyl group and feruloyl group. The identification of peak 8 was cyanidin‐3‐O‐feruloyl glucoside‐5‐O‐glucoside. The fragment pattern of peak 9 matched cyanidin‐3‐O‐sophoroside. The molecular ion of peak 11 was observed at m/z 641.17, with the characteristic fragment ions at m/z 479.12 and 317.07, which lost the hexosyl group twice based on parent ion, so it was identified as petunin‐3, 5‐O‐dihexoside. It is reported that acylated paeoniflorin glycoside and cyanidin glycoside are major anthocyanin compounds (Li et al., 2019), which was further confirmed in the present study. It was believed that anthocyanin acylated or methylated is more stable structures.

**TABLE 3:** Identification of main peaks for anthocyanin components.

Peak	Charge‐to‐mass ratio (m/z)	Molecular fragment (m/z)	Loss fragment (m/z)	Compounds	Molecular formula
4	625.18	463.12, 301.07	162	Paeoniflorin‐3, 5‐O‐dihexoside	C_28_H_33_O_16_
7	817.22	655.17	162	Petunin‐3‐O‐feruloyl‐glucoside 5‐O‐glucoside	C_38_H_41_O_20_
8	787.21	625.17, 449.11, 287.06	162, 176	Cyanidin‐3‐O‐feruloyl glucoside‐5‐O‐glucoside	C_37_H_39_O_19_
9	627.16	287.06	324	Cyanidin‐3‐O‐sophoroside	C_27_H_31_O_16_
11	641.17	479.12, 317.07	162	Petunin‐3, 5‐O‐dihexoside	C_28_H_33_O_17_

### 3.6 Antioxidant activity comparison in vitro

**FIGURE 4:**
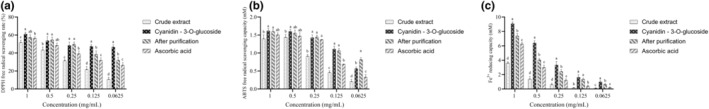
(a) The comparison between anthocyanin (before and after purification) and ascorbic acid, cyanidin‐3‐O‐glucoside on scavenging rate of DPPH free radical at different concentrations. (b) The comparison between anthocyanin (before and after purification) and ascorbic acid, cyanidin‐3‐O‐glucoside on scavenging rate of ABTS free radical at different concentrations. (c) The comparison between anthocyanin (before and after purification) and ascorbic acid, cyanidin‐3‐O‐glucoside on Fe3+ reducing capacity at different concentrations. All data are presented as mean ± SD. ^a, b, c^Within the same concentration, there is no statistical difference among the same letter (*p* < .05).

In general, as shown in Figure 4a, with the decrease in concentration, the scavenging rate of DPPH radical decreased. At the same concentration, the scavenging capacity of DPPH of anthocyanin after purification was significantly stronger than that of crude extract (*p* < .05). The scavenging rate of purified anthocyanin is significantly lower than that of cyanidin‐3‐O‐glucoside under the concentration of 0.125 and 0.0625 mg/mL (*p* < .05). The free radical DPPH is a convenient, inexpensive method for evaluating the capacity of compounds to scavenge free radicals and hydrogen suppliers. Normally, the DPPH radical is a dark purple hue in solution, but it will turn colorless or light yellow when the DPPH radical was transformed into DPPH‐H (Sridhar & Charles, 2019). A study reported that DPPH free radicals could be scavenged by polyphenols (Baliga et al., 2003) and anthocyanin happens to be a type of polyphenolic compound as well. As shown in Figure 4b, compared with the purified anthocyanin, the cyanidin‐3‐O‐glucoside of ABTS free radical scavenging capacity is significantly lower under the concentration of 0.0625 mg/mL (*p* < .05) and the crude extract of anthocyanin of ABTS free radical scavenging capacity always is significantly lower than that of purified anthocyanin (*p* < .05). Overall, ABTS free radical scavenging capacity of four antioxidants decreased, with the decrease in concentration. ABTS, as the parent substrate of ABTS free radicals reduced by antioxidants, was formed when ABTS^·+^ incubated with certain polyphenols (Ilyasov et al., 2020). The ABTS^+^ method boasts many advantages, including reacting rapidly with antioxidant substances (Walker & Everette, 2009) and less affected by pH (Zheng et al., 2016). Hence, the ABTS free radical scavenging capacity was also determined as an antioxidant activity index in vitro. Figure 4c shows the evaluation results of Fe^3+^ reducing capacity. The Fe^3+^ reducing capacity of anthocyanin after purification is significantly lower than cyanidin‐3‐O‐glucoside except at the concentration of 0.125 mg/mL (*p* < .05), whereas significantly higher than ascorbic acid and crude extract (*p* < .05). The Fe^3+^ reducing capacity was concerned since most eukaryotic organisms reduce Fe^3+^ to Fe^2+^ before taking it up (Anderson et al., 1992; Barrand et al., 1990; Wien & Van Campen, 1994). In this study, our results indicated that anthocyanin possesses favorable antioxidative activity and purification could improve its antioxidant activity. In addition, compared with the other sources of anthocyanin, anthocyanin from purple yam was almost acylated or otherwise modified, which increase anthocyanin stability greatly and agree with the previous study, but, herein, major anthocyanins from purple yams include alatanins (Moriya et al., 2015), not found in present study and differences in the stage of harvest and climate may account for this. Purple yam boasts broad development prospect due to excellent antioxidant capacity and easy preservation property.

### 3.7 Effect of purified anthocyanin on serum antioxidant capacity

**FIGURE 5:**
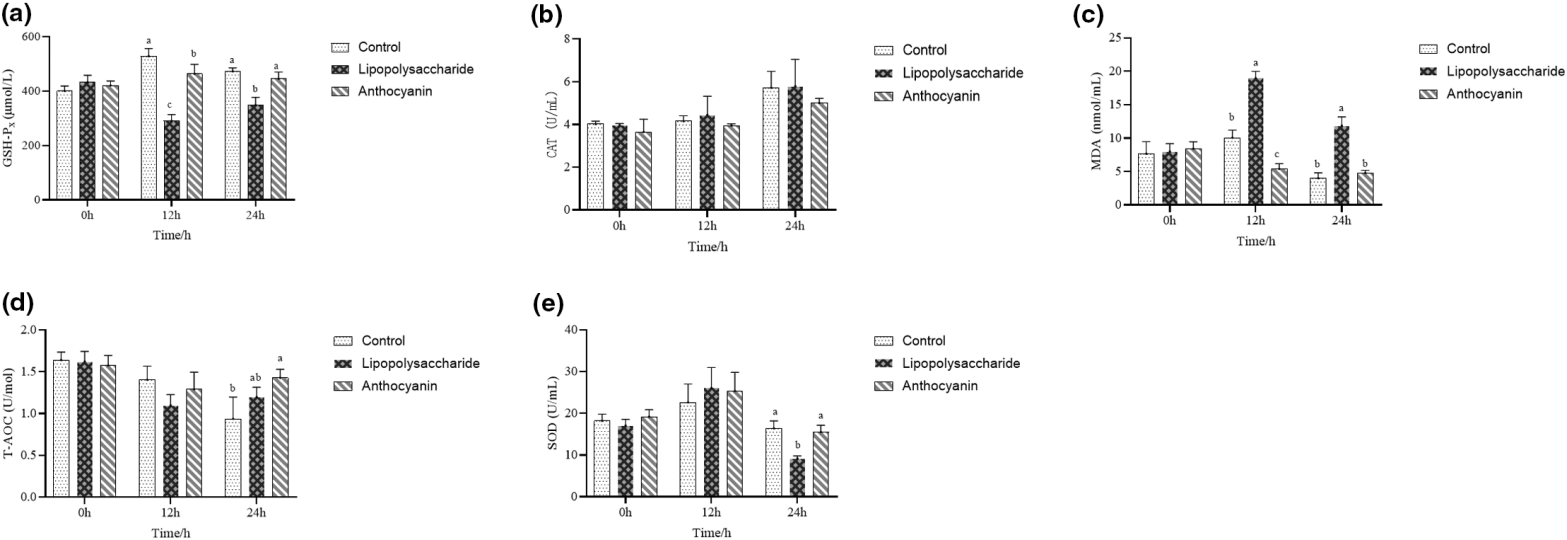
Effect of anthocyanin after purification on serum antioxidant capacity in mice at different time points. (a) GSH‐Px activity in mice serum. (b) CAT activity in mice serum. (c) MDA concentration in mice serum. (d) T‐AOC in mice serum. (e) superoxide dismutase activity in mice serum. All data are presented as mean ± SD. ^a, b, c^Within the same time point, there is no statistical difference among the same letter (*p* < .05).

The previous research found that the total antioxidant capacity (T‐AOC), antioxidant enzyme activities, and antioxidant capacity of lipoproteins could be enhanced after intaking anthocyanin (Sozański et al., 2016). In our study, compared to lipopolysaccharide group, anthocyanin significantly improved glutathione peroxidase (GSH‐P_x_) activity at 12 h and 24 h (*p* < .05) (Figure 5a). It was believed that GSH‐P_x_ plays a key role in degrading hydrogen peroxide to H_2_O and alcohols in stress status (Bela et al., 2015). Niki E et al. also found that vascular endothelial adhesion molecule and monocyte chemotactic protein decreased, with the increase in GSH‐Px activity (Niki & Transactions, 2004). Herein, acute oxidative stress lipopolysaccharide‐challenged decreased GSH‐Px activity, whereas anthocyanin reversed the negative effect. Catalase (CAT), one of the four antioxidant enzymes, protects antioxidant system against oxidative stress with decomposing hydrogen peroxide (Li et al., 2018) and there was no significant differences in our study (*p* > .05) (Figure 5b). As shown in Figure 5c, anthocyanin significantly decreased malondialdehyde (MDA) concentration on serum at 12 h and 24 h (*p* < .05), compared with lipopolysaccharide group. MDA, product of lipid peroxidation, reflects oxidative stress extents (Giera et al., 2012). Our results suggested that anthocyanin effectively inhibited lipid peroxidation induced by lipopolysaccharide. Figure 5d shows that, compared with lipopolysaccharide group, there was no significant effect of anthocyanin on T‐AOC (*p* > .05). Superoxide dismutase (SOD) plays a critical role in controlling the levels of extracellular superoxide (Oury et al., 1996). In this research, we found that anthocyanin significantly increased SOD activity at 24 h after the decrease that was induced by lipopolysaccharide (*p* < .05) (Figure 5e). Production and clearance of reactive oxygen species (ROS) is a relative equilibrium status. However, overproduction of radical species damage certain cell components and augment pathogenesis (Lundgren et al., 2018). The enzymatic antioxidants can counteract radicals due to the property to degrade ROS in body (He et al., 2017) and endogenous antioxidants, including SOD, CAT, and GSH‐Px play irreplaceable roles. Herein, our results suggested that anthocyanin extracted from DL ameliorated antioxidant system, reversed the oxidative damage lipopolysaccharide‐challenged.

## 4 CONCLUSION

In this study, we purified anthocyanin from DL using three successive steps, which enhanced the purity of anthocyanin. The identification of UPLC‐MS analysis for anthocyanin after purified by LH‐20 Sephadex suggested that there were five anthocyanins, in which petunin‐3‐O‐feruloyl‐glucoside‐5‐O‐glucoside accounts for 76.77%. Furthermore, the result of antioxidant capacity evaluation indicated that anthocyanin possessed favorable antioxidant ability in vitro and had a positive protective effect against oxidative attack in vivo after purification. In summary, anthocyanin from purple yam not only possess a stable chemical structure conducive to store, but also has excellent antioxidant ability both in vivo and in vitro.

## AUTHOR CONTRIBUTIONS

Pingfei Qiu: Writing – original draft, Writing – review & editing. Junpu Chen: Conceptualization. Junlong Wu: Software. Qin Wang: Validation. Yanrong Hu: Methodology. Xiaochun Li: Resources. Huiyu Shi: Project administration. Xuemei Wang: Funding acquisition, supervision.

## FUNDING INFORMATION

This work was financed by the grants from the Innovative Research Projects of Education Department of Hainan Province (Grant No. Qhys2021‐164) and the National Natural Science Foundation of China (Grant No. 31960677).

## CONFLICT OF INTEREST STATEMENT

The authors declare that the research was conducted in the absence of any commercial or financial relationships that could be construed as a potential conflict of interest.

## Supporting information


Data S1.

## Data Availability

The data that support the findings of this study are available from the corresponding author on request.
