# Supplementary figures and images for: The effect of anthocyanin from Dioscorea alata L. after purification, identification on antioxidant capacity in mice
Source: Food Sci Nutr. 2023 Jul 18;11(10):6106–15. doi: 10.1002/fsn3.3547 (PMC10563728; doi:10.1002/fsn3.3547)

RT: 0.00 - 26.01

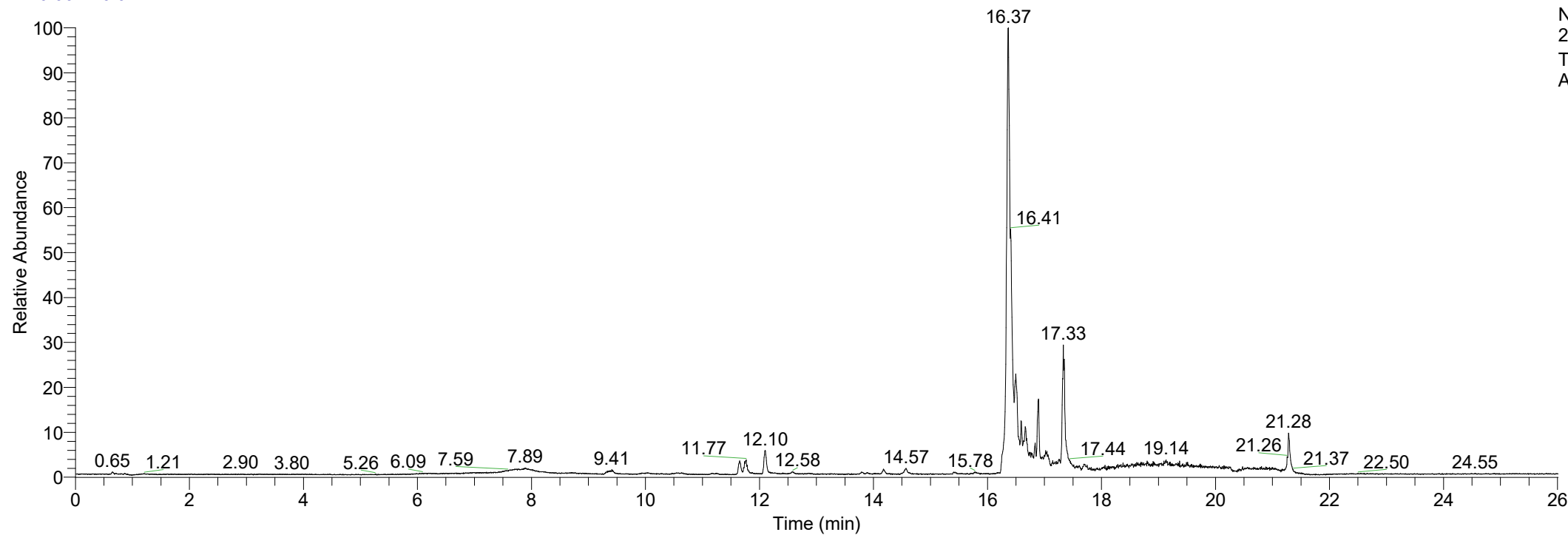

NL:  
2.85E7  
TIC MS  
Anthy\_1

Anthy\_1 #1 RT: 0.01 AV: 1 NL: 5.85E4

T: FTMS + p ESI SIM msx ms [269.0601-273.0601, 285.0550-289.0550, 300.0000-304.0000, 315.0656-319.0656, 329.0812-333.0812, 447.1078-451.1078, 461.1235-465.1235, 577.1497-581.1497, 6 ...

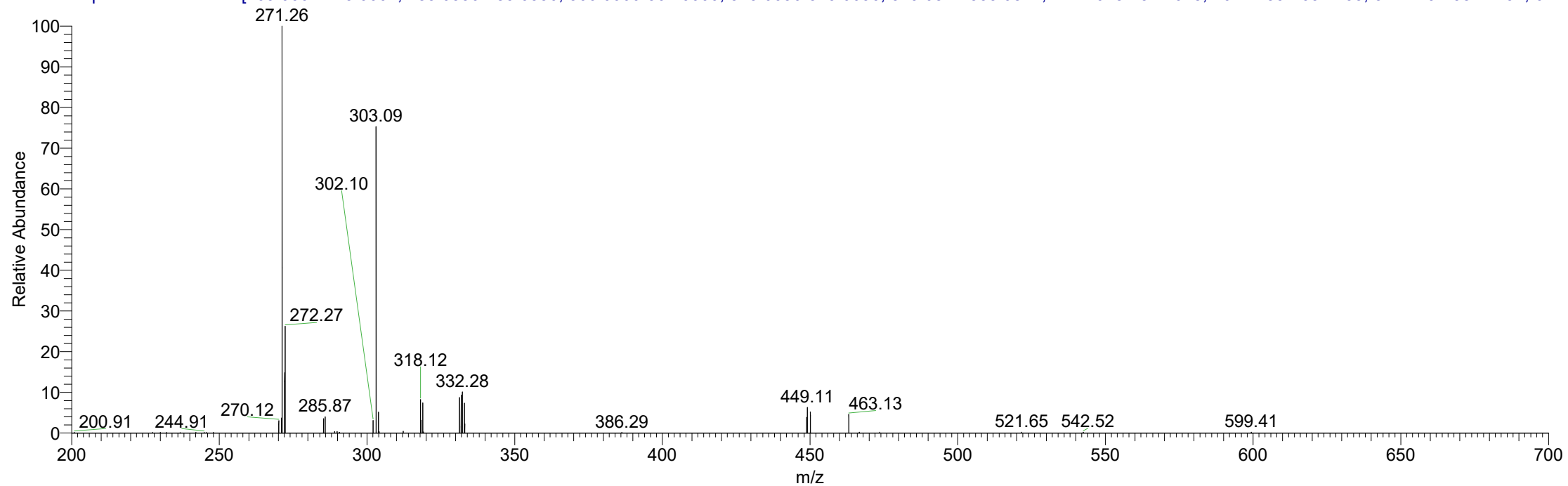

Supplement: Supplementary file 1 — Data S1. [file FSN3-11-6106-s001.zip › 1.pdf]

RT: 0.00 - 26.01

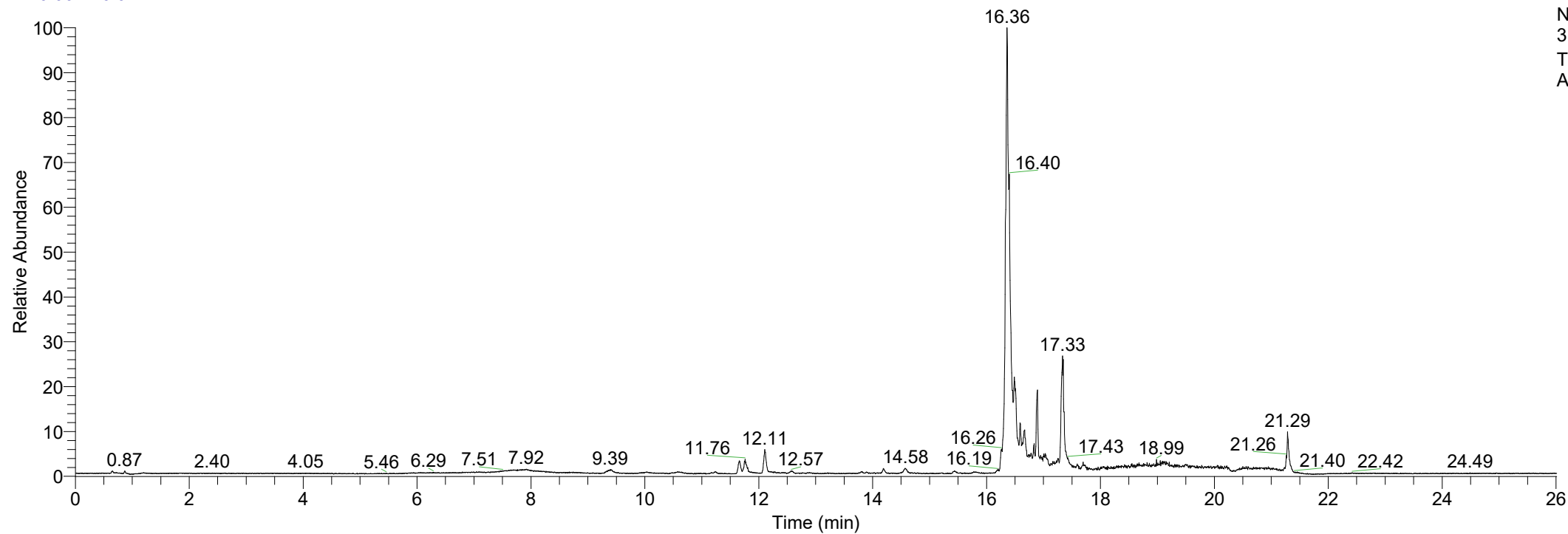

Anthy\_10 #1 RT: 0.01 AV: 1 NL: 5.36E4

T: FTMS + p ESI SIM msx ms [269.0601-273.0601, 285.0550-289.0550, 300.0000-304.0000, 315.0656-319.0656, 329.0812-333.0812, 447.1078-451.1078, 461.1235-465.1235, 577.1497-581.1497, 6 ...

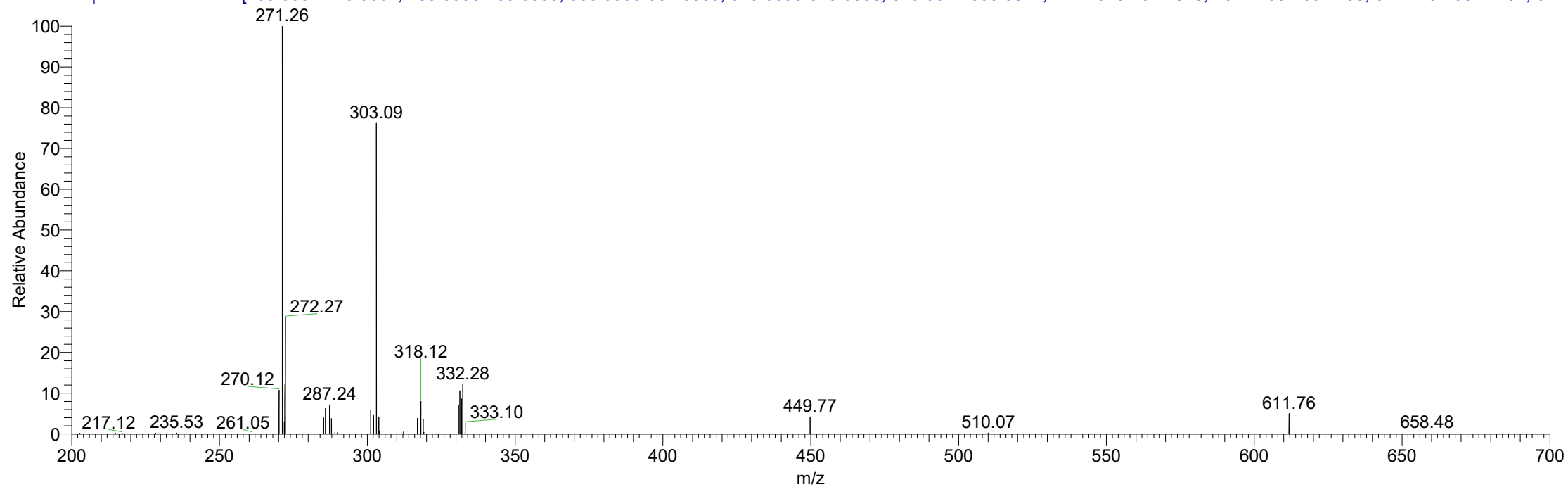

Supplement: Supplementary file 1 — Data S1. [file FSN3-11-6106-s001.zip › 10.pdf]

RT: 0.00 - 26.01

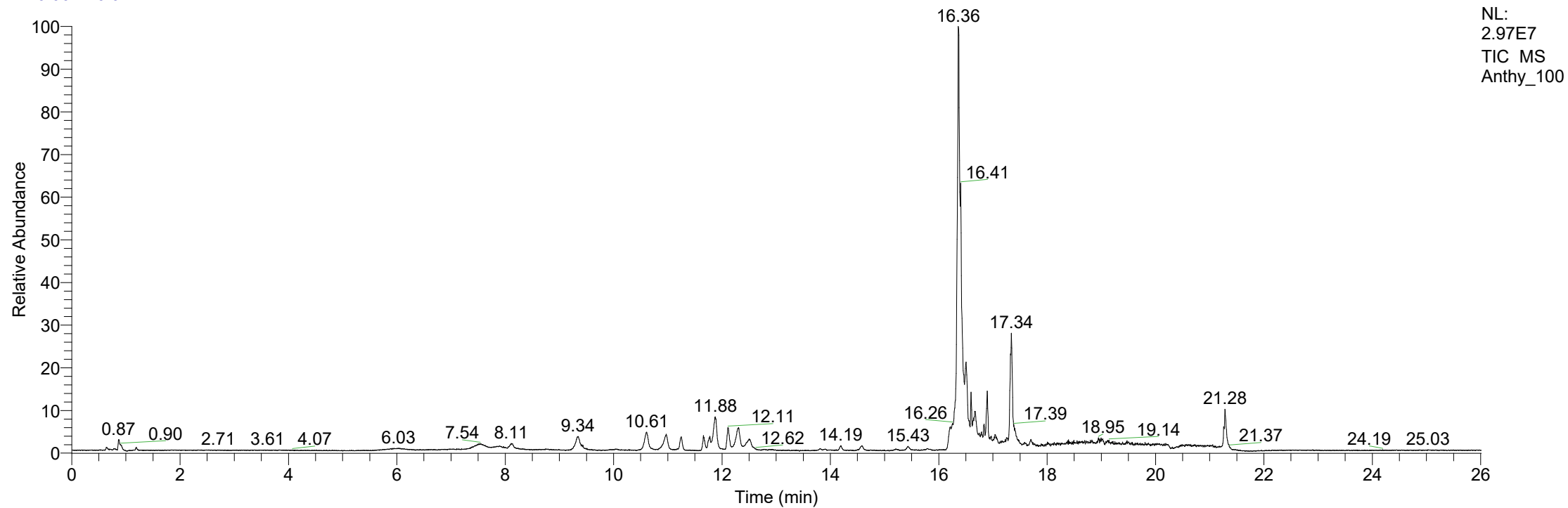

Anthy\_100 #1 RT: 0.01 AV: 1 NL: 5.92E4

T: FTMS + p ESI SIM msx ms [269.0601-273.0601, 285.0550-289.0550, 300.0000-304.0000, 315.0656-319.0656, 329.0812-333.0812, 447.1078-451.1078, 461.1235-465.1235, 577.1497-581.1497, 6 ...

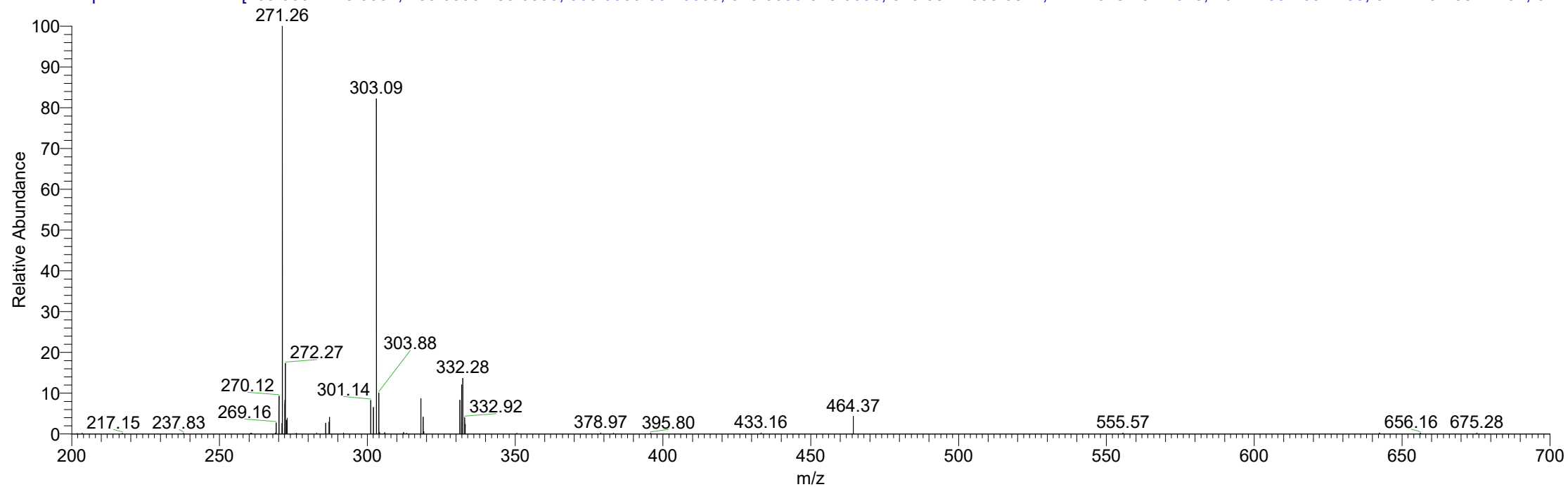

Supplement: Supplementary file 1 — Data S1. [file FSN3-11-6106-s001.zip › 100.pdf]

RT: 0.00 - 26.01

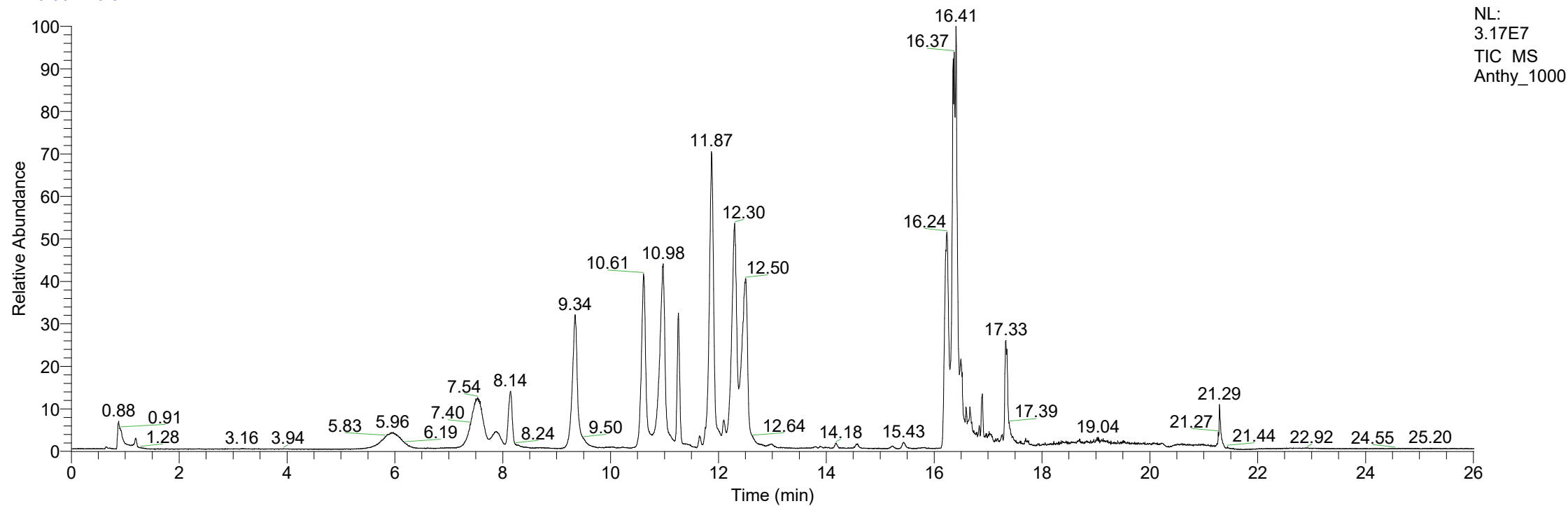

Anthy\_1000 #1 RT: 0.01 AV: 1 NL: 5.97E4

T: FTMS + p ESI SIM msx ms [269.0601-273.0601, 285.0550-289.0550, 300.0000-304.0000, 315.0656-319.0656, 329.0812-333.0812, 447.1078-451.1078, 461.1235-465.1235, 577.1497-581.1497, 6 ...

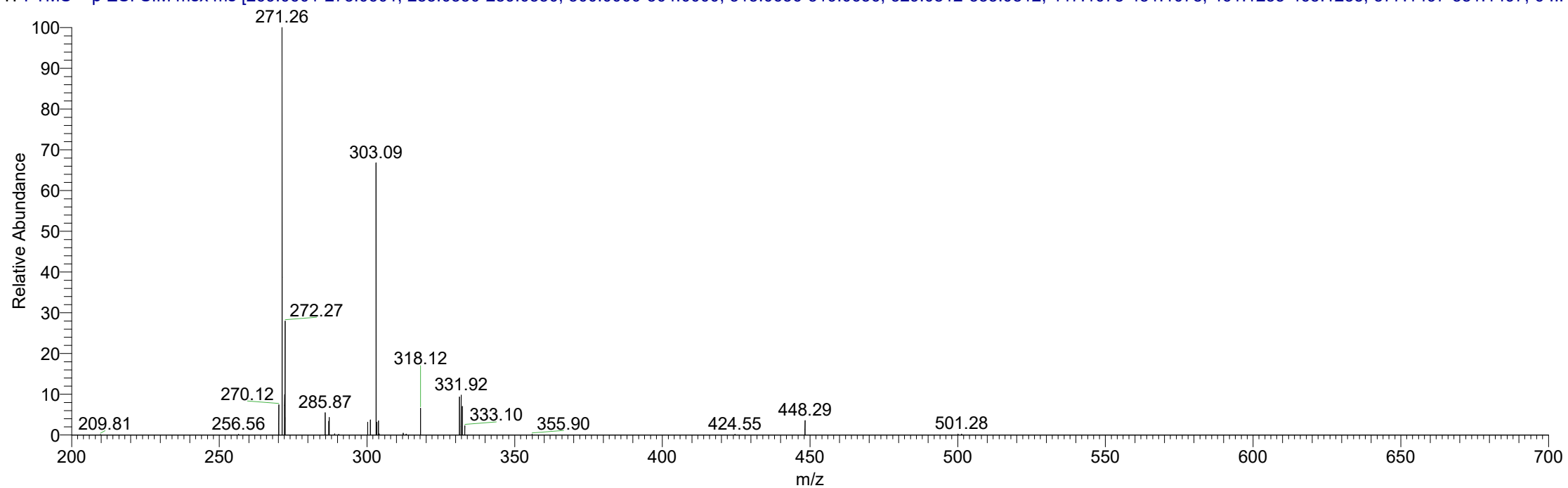

Supplement: Supplementary file 1 — Data S1. [file FSN3-11-6106-s001.zip › 1000.pdf]

RT: 0.00 - 26.01

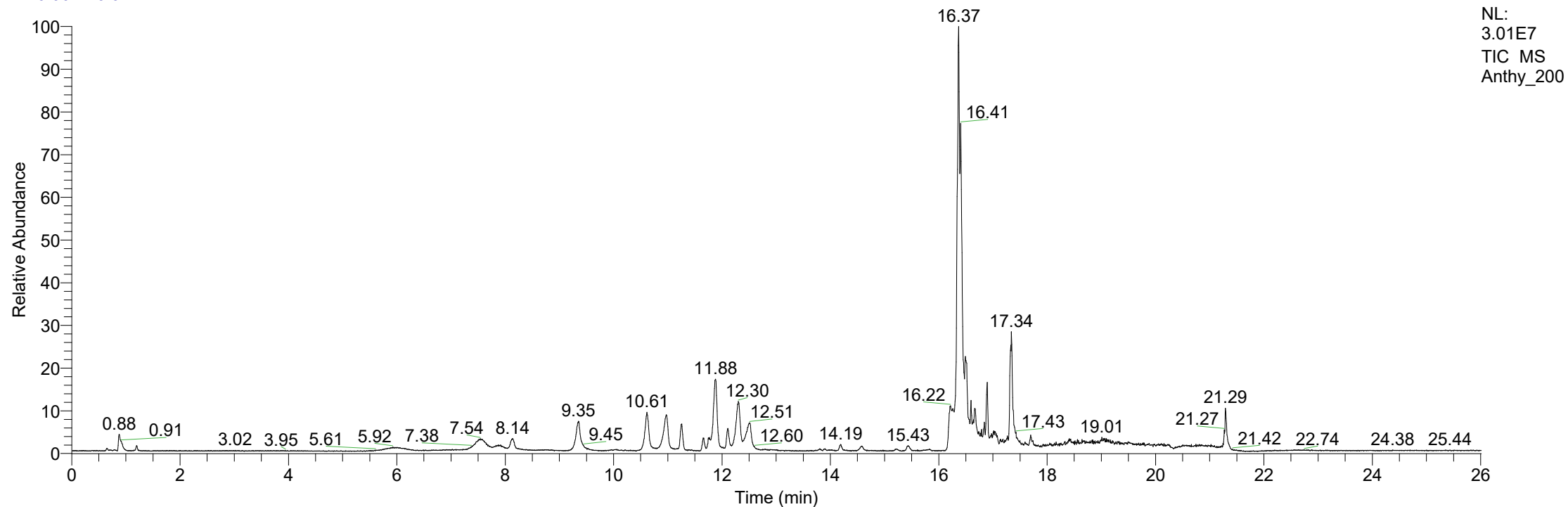

Anthy\_200 #1 RT: 0.01 AV: 1 NL: 6.69E4

T: FTMS + p ESI SIM msx ms [269.0601-273.0601, 285.0550-289.0550, 300.0000-304.0000, 315.0656-319.0656, 329.0812-333.0812, 447.1078-451.1078, 461.1235-465.1235, 577.1497-581.1497, 6 ...

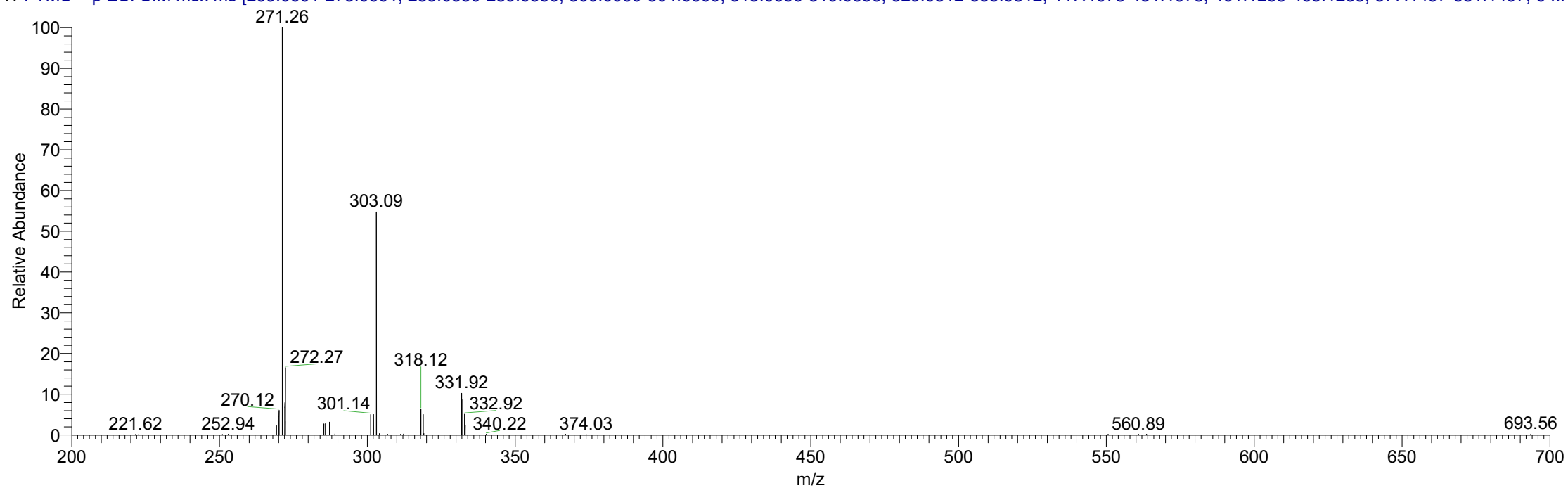

Supplement: Supplementary file 1 — Data S1. [file FSN3-11-6106-s001.zip › 200.pdf]

RT: 0.00 - 26.01

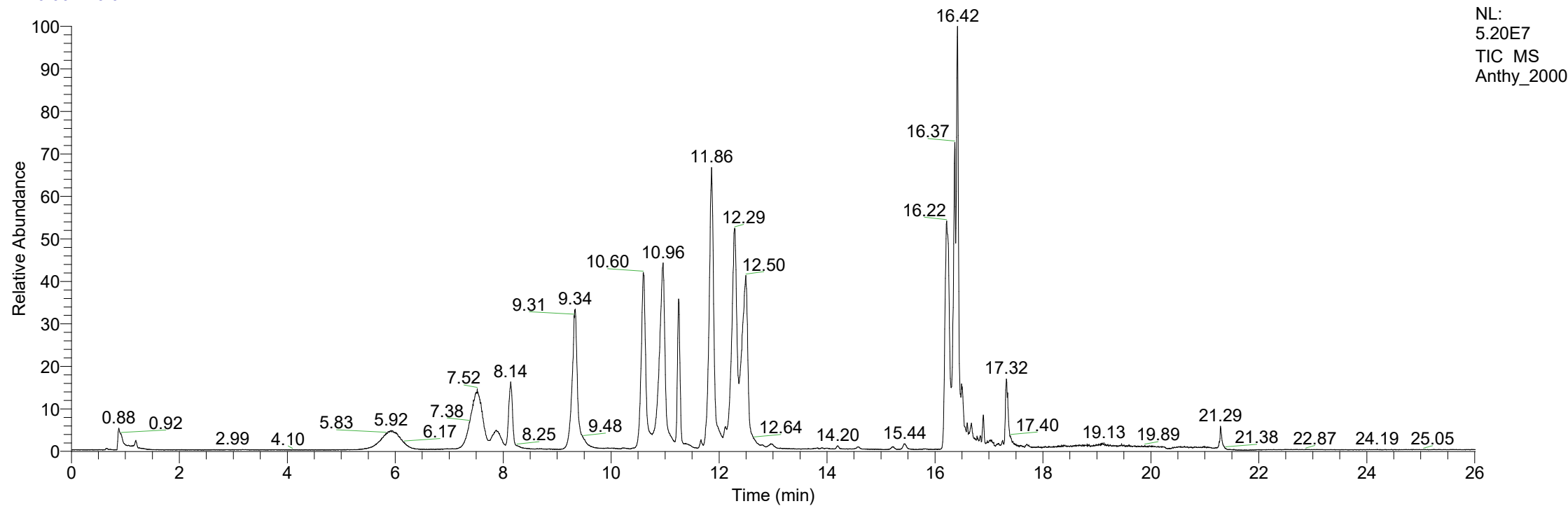

Anthy\_2000 #1 RT: 0.01 AV: 1 NL: 6.24E4

T: FTMS + p ESI SIM msx ms [269.0601-273.0601, 285.0550-289.0550, 300.0000-304.0000, 315.0656-319.0656, 329.0812-333.0812, 447.1078-451.1078, 461.1235-465.1235, 577.1497-581.1497, 6 ...

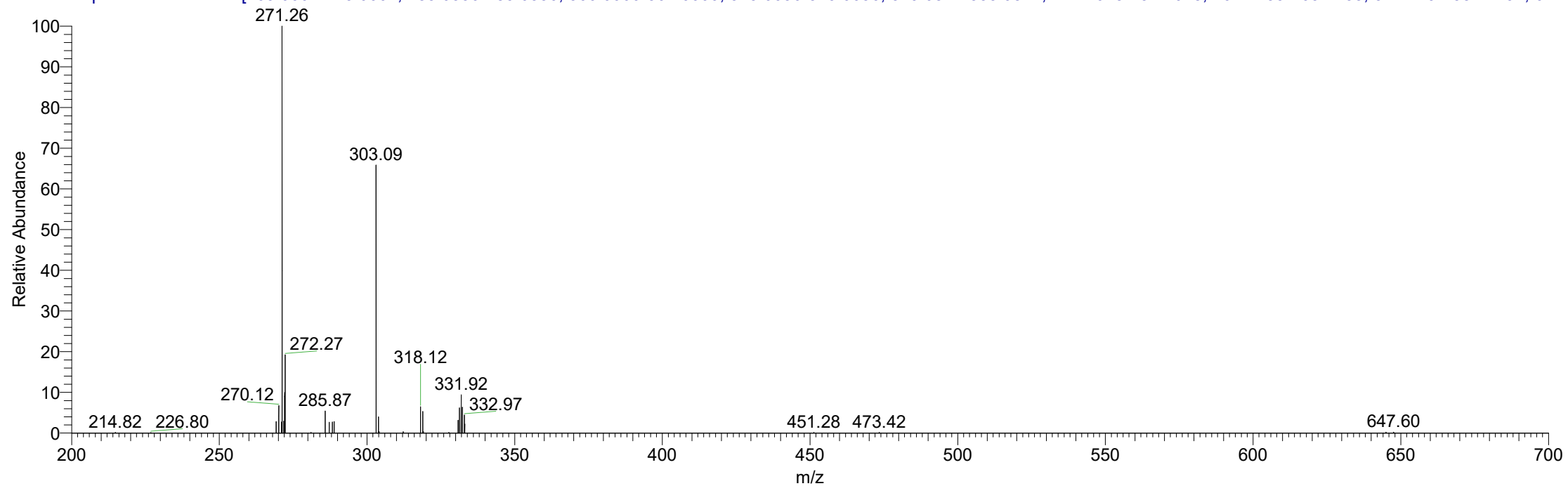

Supplement: Supplementary file 1 — Data S1. [file FSN3-11-6106-s001.zip › 2000.pdf]

RT: 0.00 - 26.01

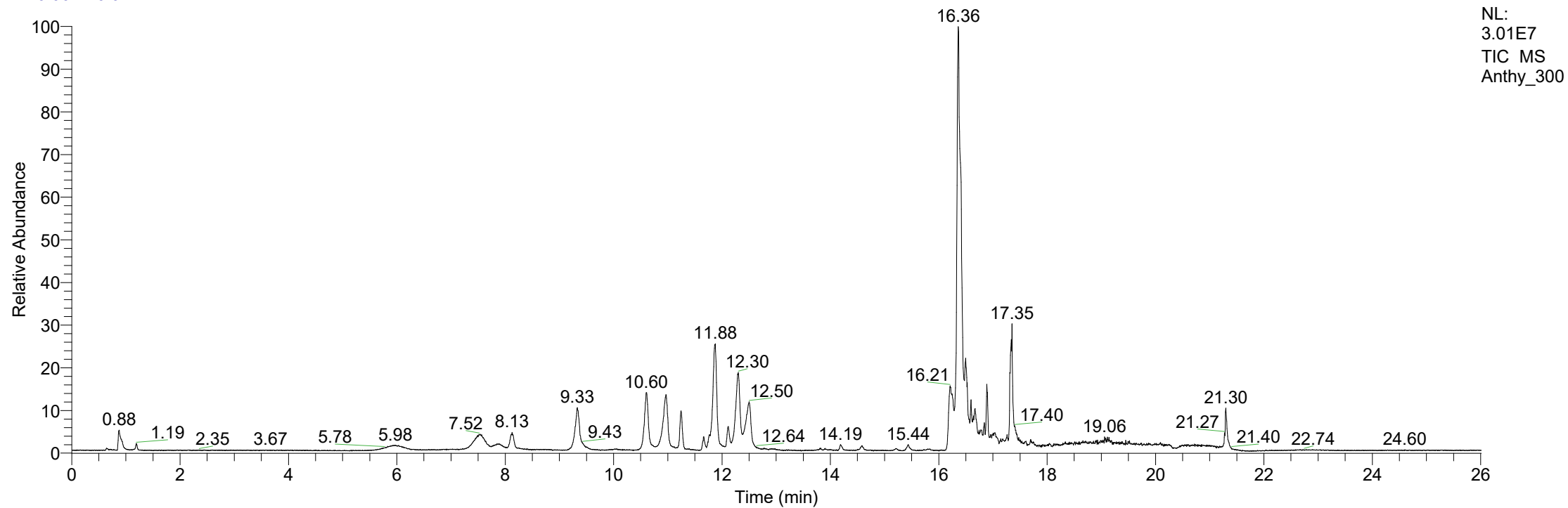

Anthy\_300 #1 RT: 0.01 AV: 1 NL: 7.39E4

T: FTMS + p ESI SIM msx ms [269.0601-273.0601, 285.0550-289.0550, 300.0000-304.0000, 315.0656-319.0656, 329.0812-333.0812, 447.1078-451.1078, 461.1235-465.1235, 577.1497-581.1497, 6 ...

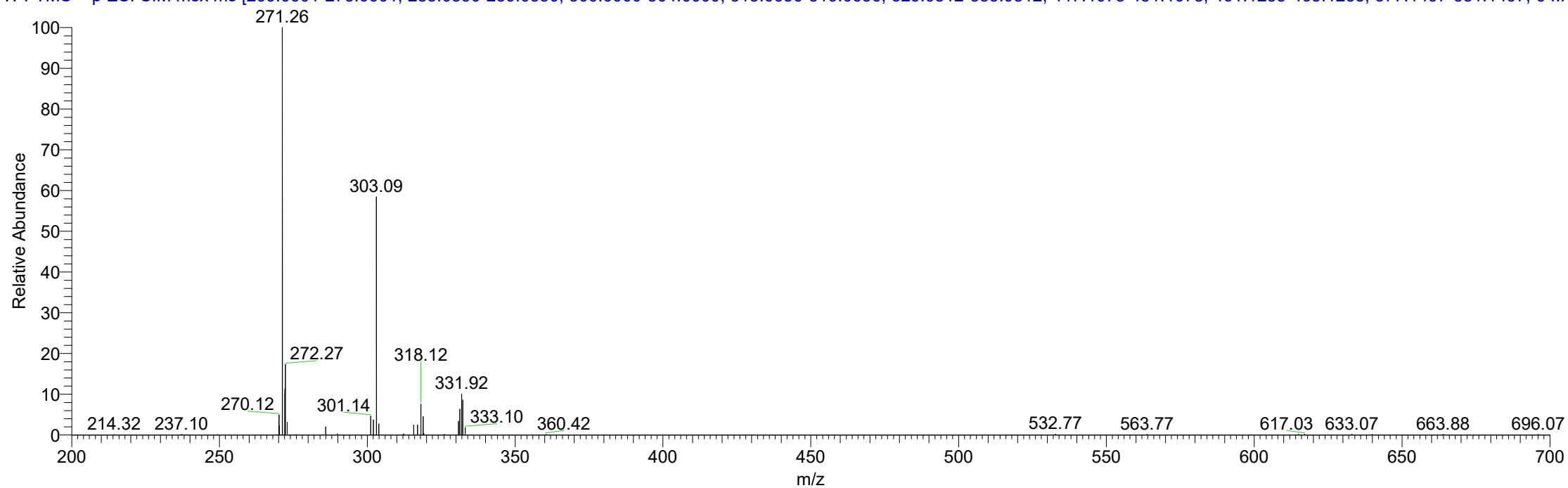

Supplement: Supplementary file 1 — Data S1. [file FSN3-11-6106-s001.zip › 300.pdf]

RT: 0.00 - 26.01

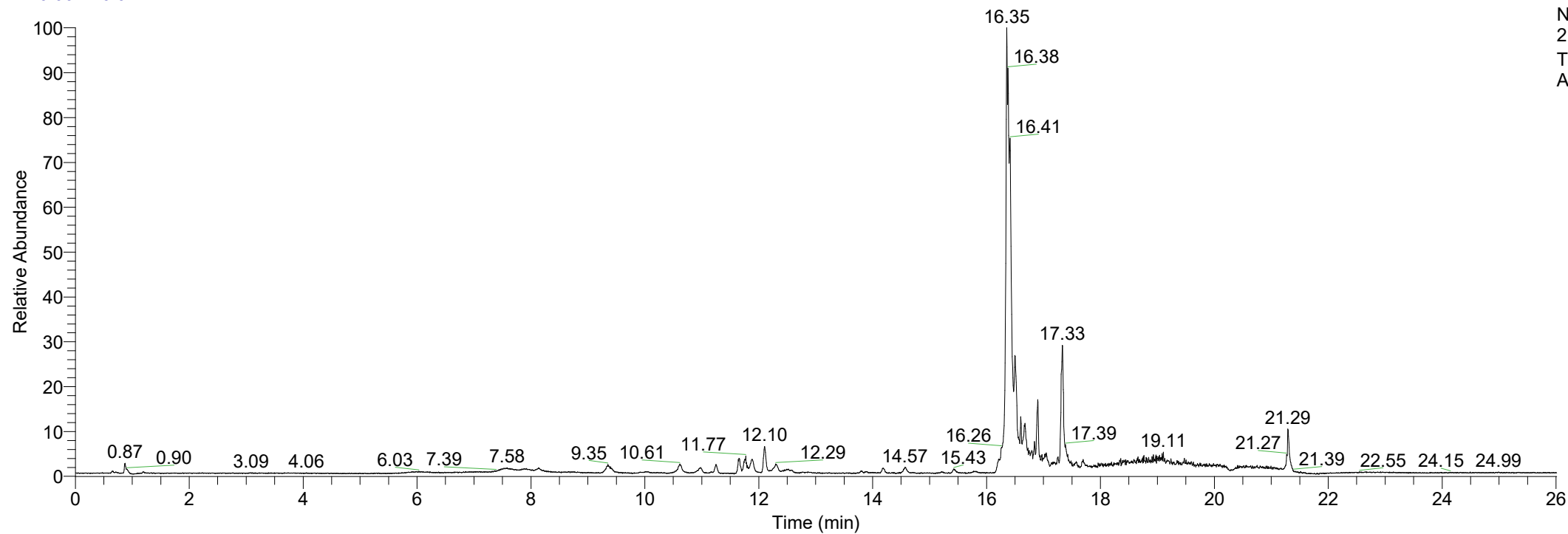

NL:  
2.65E7  
TIC MS  
Anthy\_50

Anthy\_50 #1 RT: 0.01 AV: 1 NL: 7.00E4

T: FTMS + p ESI SIM msx ms [269.0601-273.0601, 285.0550-289.0550, 300.0000-304.0000, 315.0656-319.0656, 329.0812-333.0812, 447.1078-451.1078, 461.1235-465.1235, 577.1497-581.1497, 6 ...

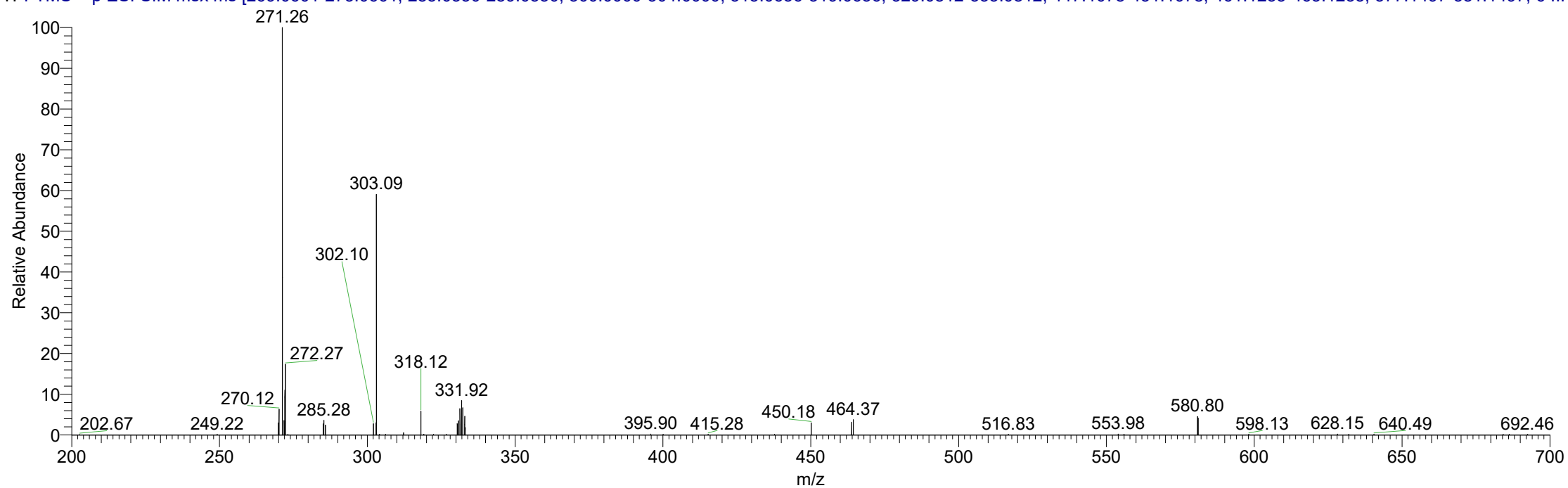

Supplement: Supplementary file 1 — Data S1. [file FSN3-11-6106-s001.zip › 50.pdf]

RT: 0.00 - 26.01

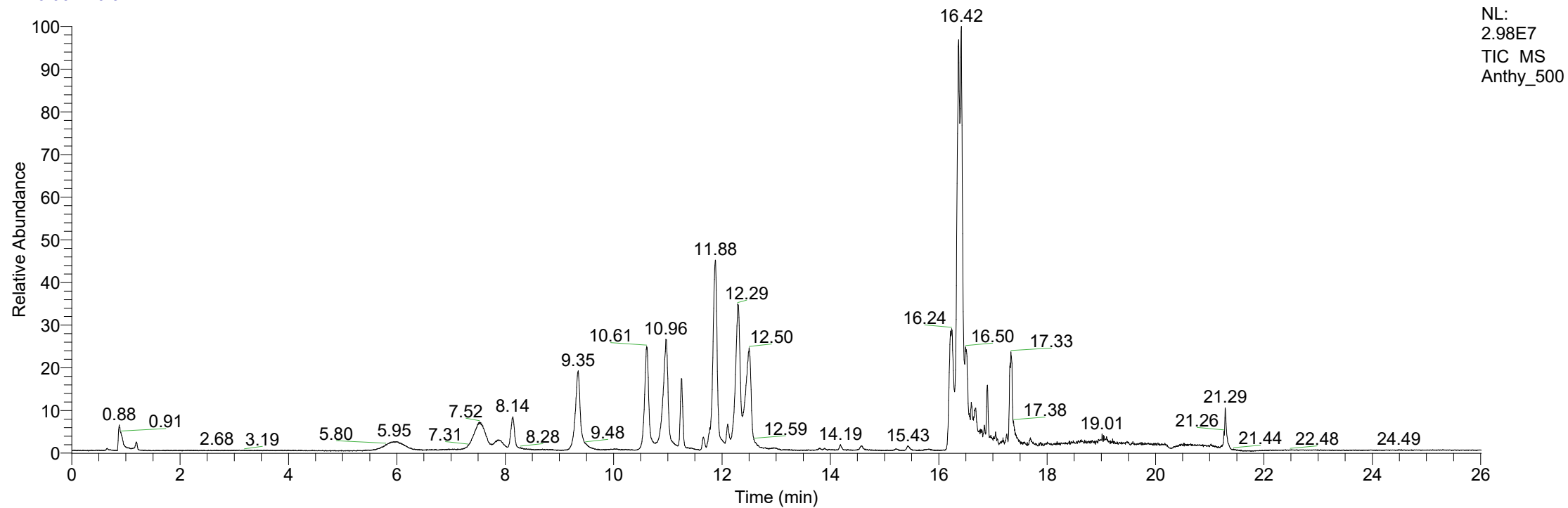

Anthy\_500 #1 RT: 0.01 AV: 1 NL: 5.84E4

T: FTMS + p ESI SIM msx ms [269.0601-273.0601, 285.0550-289.0550, 300.0000-304.0000, 315.0656-319.0656, 329.0812-333.0812, 447.1078-451.1078, 461.1235-465.1235, 577.1497-581.1497, 6 ...

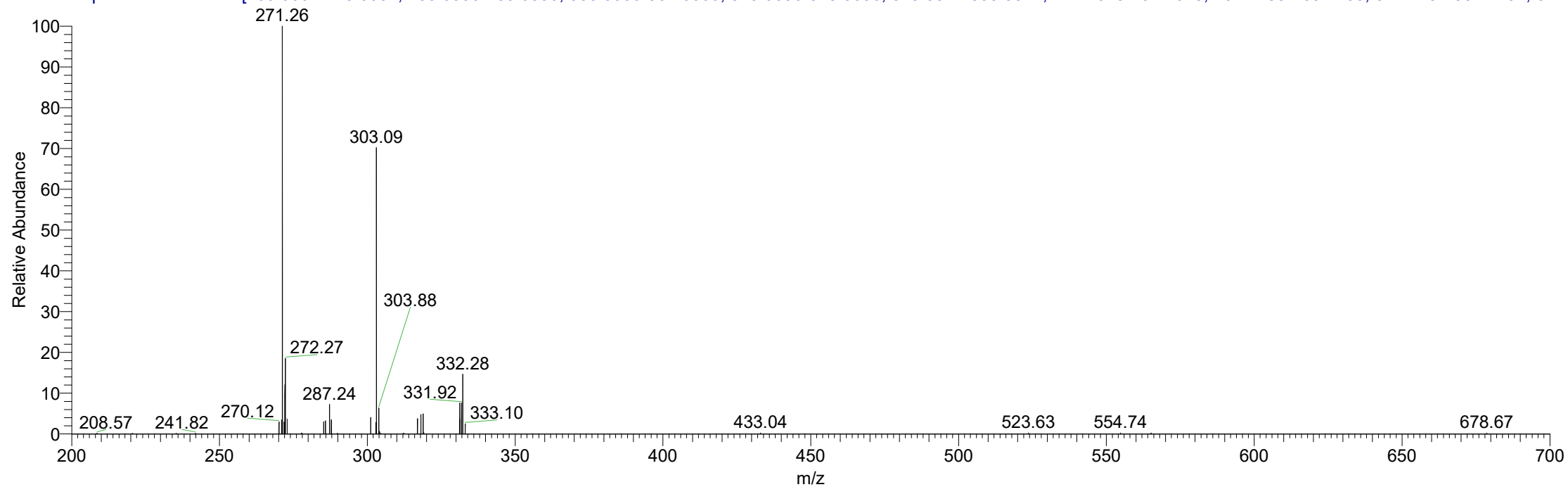

Supplement: Supplementary file 1 — Data S1. [file FSN3-11-6106-s001.zip › 500.pdf]

RT: 0.00 - 26.01

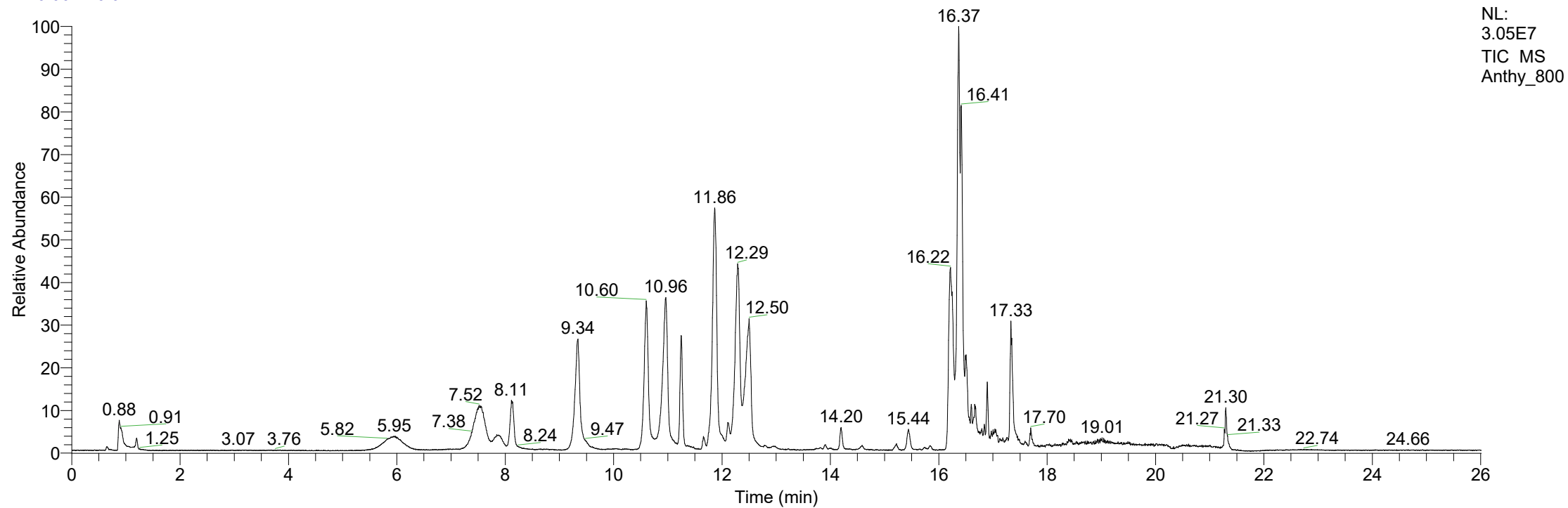

Anthy\_800 #1 RT: 0.01 AV: 1 NL: 6.74E4

T: FTMS + p ESI SIM msx ms [269.0601-273.0601, 285.0550-289.0550, 300.0000-304.0000, 315.0656-319.0656, 329.0812-333.0812, 447.1078-451.1078, 461.1235-465.1235, 577.1497-581.1497, 6 ...

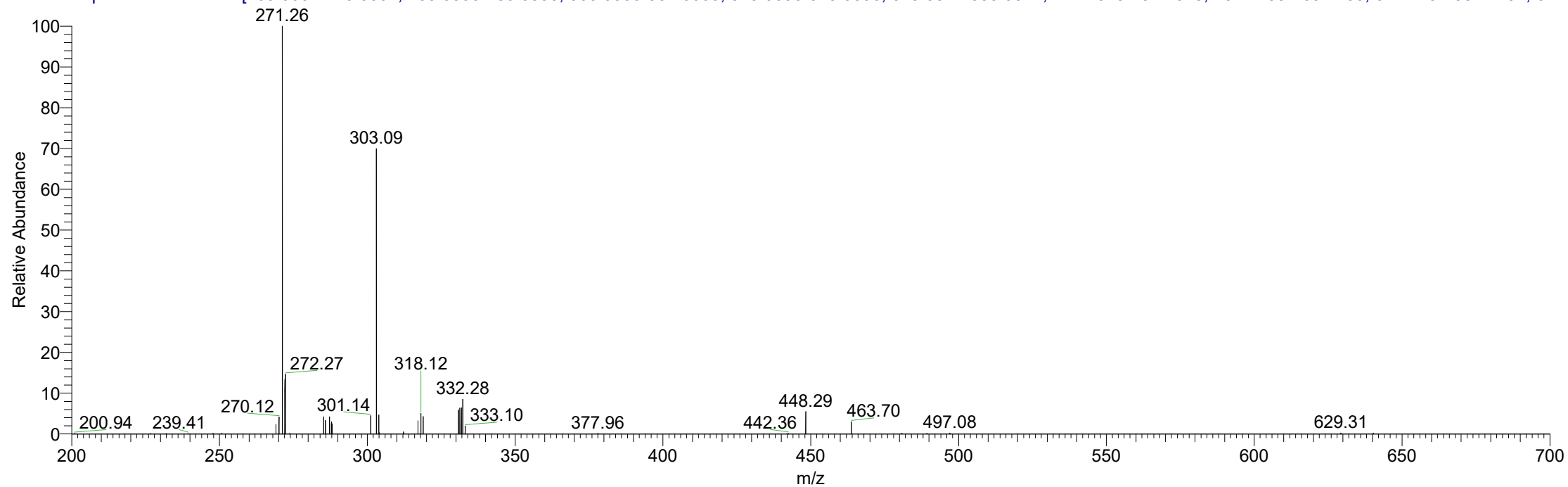

Supplement: Supplementary file 1 — Data S1. [file FSN3-11-6106-s001.zip › 800.pdf]

RT: 0.00 - 26.01

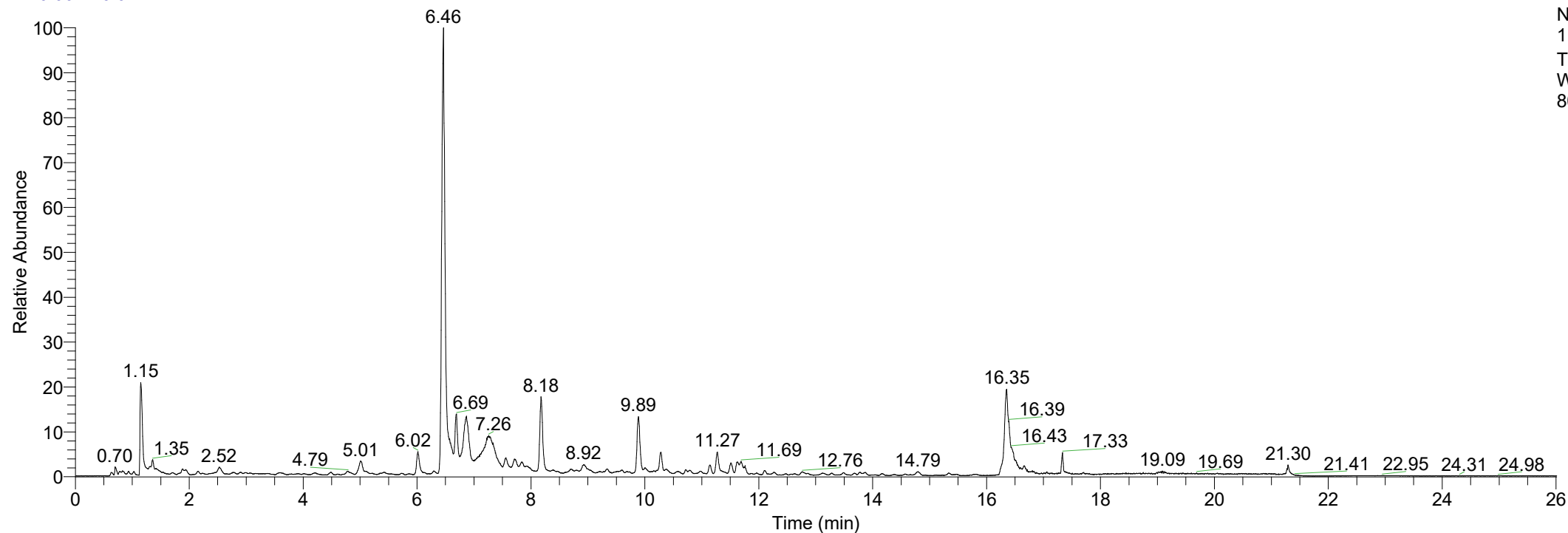

NL:  
1.17E8  
TIC MS  
WXM21010  
8001

WXM210108001 #1 RT: 0.01 AV: 1 NL: 4.64E4

T: FTMS + p ESI SIM msx ms [269.0601-273.0601, 285.0550-289.0550, 300.0000-304.0000, 315.0656-319.0656, 329.0812-333.0812, 447.1078-451.1078, 461.1235-465.1235, 577.1497-581.1497, 6 ...

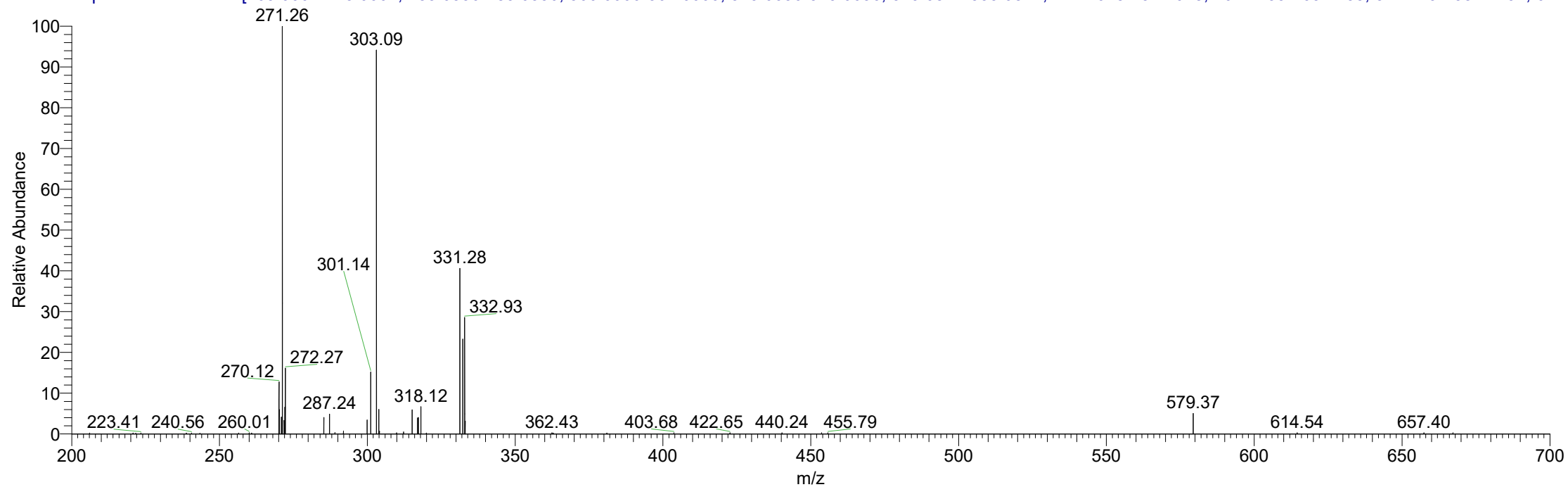

Supplement: Supplementary file 1 — Data S1. [file FSN3-11-6106-s001.zip › Samples-1.pdf]

RT: 0.00 - 26.01

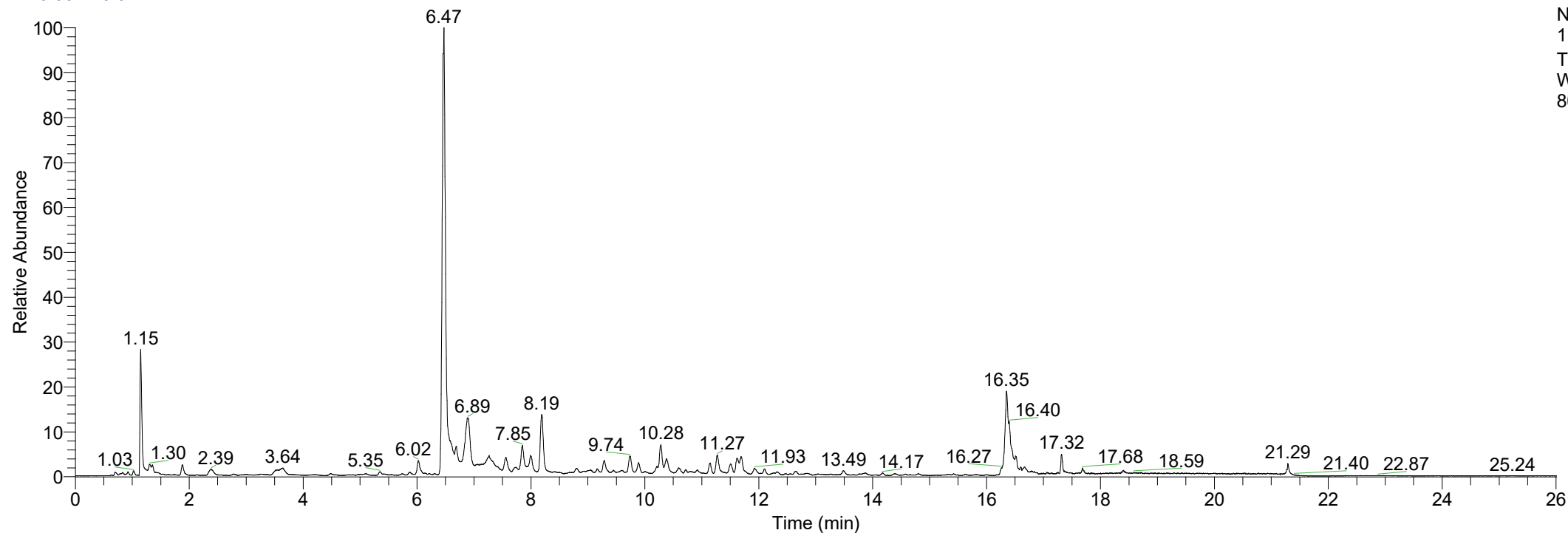

NL:  
1.08E8  
TIC MS  
WXM21010  
8002

WXM210108002 #1 RT: 0.01 AV: 1 NL: 5.11E4

T: FTMS + p ESI SIM msx ms [269.0601-273.0601, 285.0550-289.0550, 300.0000-304.0000, 315.0656-319.0656, 329.0812-333.0812, 447.1078-451.1078, 461.1235-465.1235, 577.1497-581.1497, 6 ...

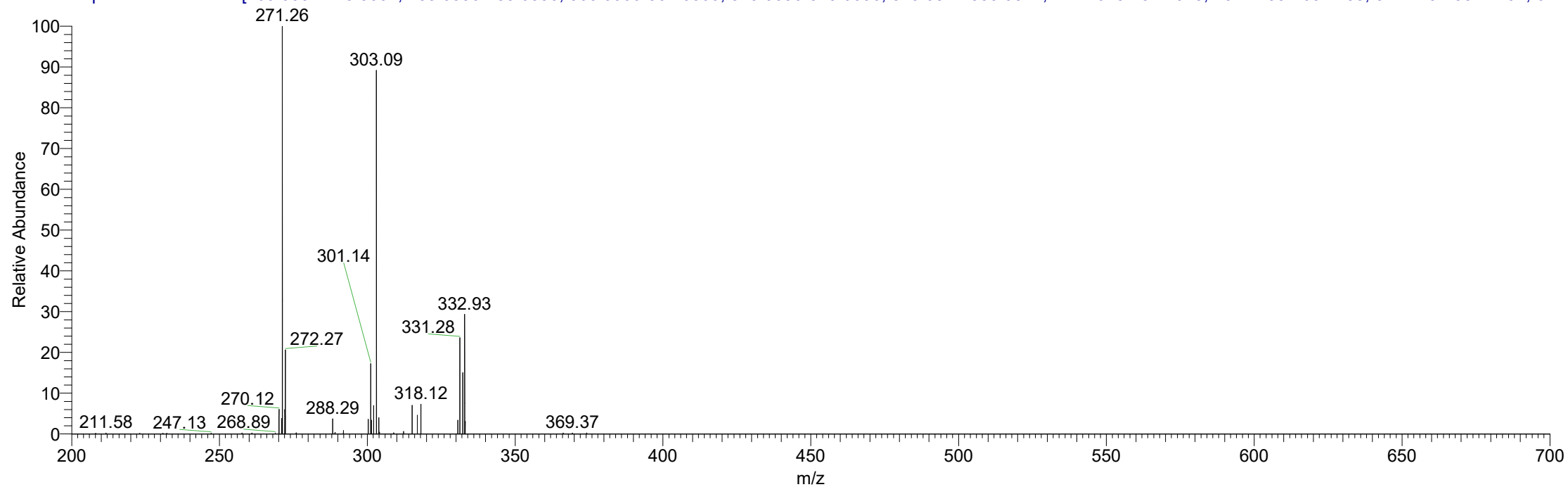

Supplement: Supplementary file 1 — Data S1. [file FSN3-11-6106-s001.zip › Samples-2.pdf]

RT: 0.00 - 26.01

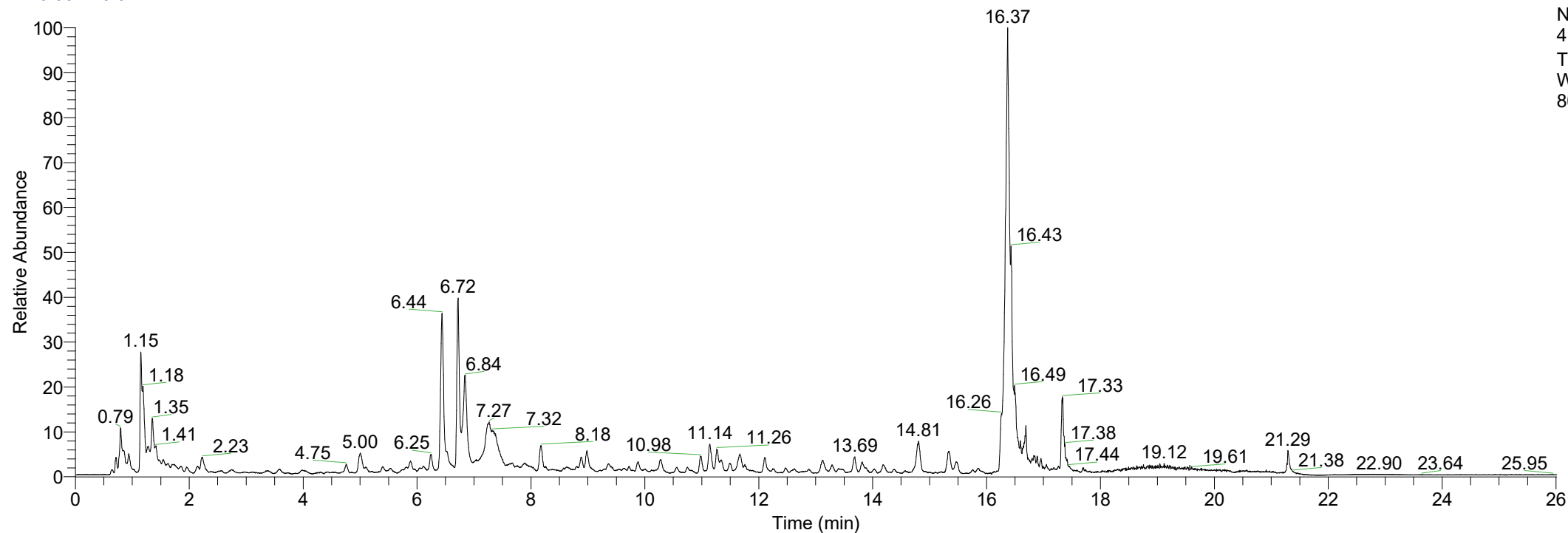

NL:  
4.51E7  
TIC MS  
WXM21010  
8003

WXM210108003 #1 RT: 0.01 AV: 1 NL: 6.03E4

T: FTMS + p ESI SIM msx ms [269.0601-273.0601, 285.0550-289.0550, 300.0000-304.0000, 315.0656-319.0656, 329.0812-333.0812, 447.1078-451.1078, 461.1235-465.1235, 577.1497-581.1497, 6 ...

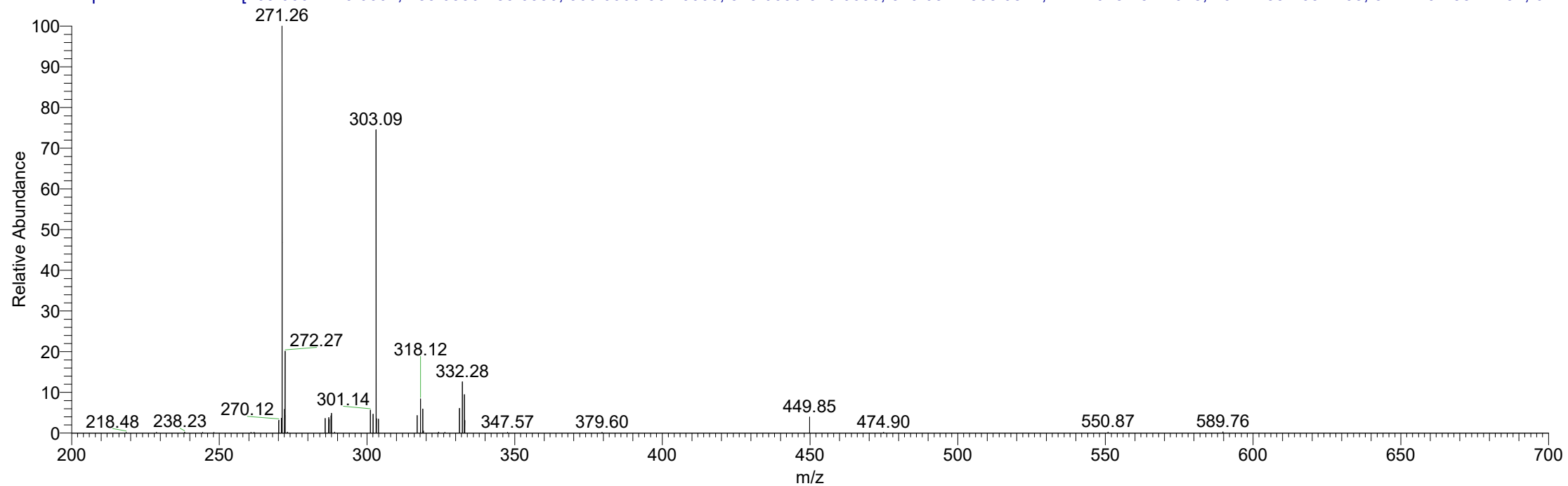

Supplement: Supplementary file 1 — Data S1. [file FSN3-11-6106-s001.zip › Samples-3.pdf]
